# Logic Models on Health Information Technology–Related Interventions: Scoping Review Across Disciplines

**DOI:** 10.2196/87848

**Published:** 2026-07-28

**Authors:** Elske Ammenwerth, Iiris Hörhammer, Michelle Bindel

**Affiliations:** 1 UMIT TIROL – Private University for Health Sciences and Health Technology, Eduard Wallnöfer Zentrum 1, Hall in Tirol, 6060, Austria; 2 Aalto University, Helsinki, Uusimaa, Finland

**Keywords:** medical informatics, program evaluation, implementation science, program theory, logic model, theory of change

## Abstract

**Background:**

Health information technology (HIT) interventions are complex, context-dependent, and often insufficiently theorized, which can hinder their design, implementation, and evaluation. Program theory approaches such as logic models and theory of change (ToC) are well-established in public health and implementation science for articulating causal assumptions. Their use in medical informatics, however, appears inconsistent. A systematic overview of how logic models and ToC have been applied to HIT interventions is therefore needed to support theory-informed development and cumulative learning in the field.

**Objective:**

We aimed to map how logic models, ToC, and related program theory approaches have been conceptualized, constructed, and applied in HIT-related interventions across disciplines. A secondary objective was to identify implications for medical informatics research and practice.

**Methods:**

Following PRISMA-ScR (Preferred Reporting Items for Systematic Reviews and Meta-Analyses extension for Scoping Reviews), searches were conducted in PubMed, Web of Science, Academic Search Elite, APA PsycArticles, and CINAHL. Eligible publications used a logic model, ToC, or related construct within an HIT intervention in any health care or social-care setting. Next, 2 reviewers independently screened records and extracted data on study characteristics, type and purpose of HIT, model structure, theoretical foundations, and reported benefits and challenges.

**Results:**

A total of 69 publications (2012‐2025) met the criteria. Use of program theory increased markedly after 2020 and spanned medical informatics, public health, health services research, and implementation science. Logic models were most frequently applied to patient-facing and self-management technologies, particularly mobile health, telehealth, and home-based remote monitoring. Most models were used to support HIT development or evaluation. Of the total, 60 (87%) studies provided a logic model visualization, although structures varied considerably. Out of 69, 50 (72%) studies cited guidelines for model development, most commonly UK Medical Research Council guidance, realist evaluation, or the Kellogg Logic Model. Out of 69 studies, 28 (41%) used behavioral or implementation frameworks, such as Capability, Opportunity, Motivation–Behavior model (COM-B), Consolidated Framework for Implementation Research (CFIR), Expert Recommendations for Implementing Change (ERIC), Fit between Individuals, Task, and Technology (FITT), or Non-adoption, Abandonment, and challenges to the Scale-up, Spread, and Sustainability (NASSS) to populate model content. Only 3 (4%) studies reused an existing model. Reported benefits concerned improved theorization, structured evaluation, and stakeholder engagement; challenges included limited empirical evidence, high resource demands, and tensions between specificity and generalizability.

**Conclusions:**

Program theory approaches are increasingly used to conceptualize and evaluate HIT interventions; yet, their application in medical informatics remains fragmented. More systematic and theory-informed use of logic models could enhance conceptual clarity, methodological rigor, and cumulative learning. Future work should promote model reuse, establish repositories, strengthen reporting standards, and integrate program theory in HIT education and research to support coherent development, evaluation, and scaling of digital health interventions.

## Introduction

### Background

Health information technology (HIT) is expected to improve the quality, efficiency, and equity in care delivery. Yet, many HIT implementations fall short of intended outcomes because these interventions are complex, context-dependent, and insufficiently theorized during their design and implementation [1]. Guidance on complex interventions emphasizes explicit theorization and iterative refinement with stakeholders across the lifecycle of development, piloting, evaluation, and scaling up [2,3].

At the core of theory-informed evaluation lies program theory—an explicit account of how and why a program is expected to produce outcomes by linking resources, activities, mechanisms, and outcomes. Program theory renders assumptions testable and provides a scaffold for both program design and evaluation [4]. A program in this perspective is “a set of organized activities or interventions supported by resources designed to achieve a specific result” [5].

The concept of program theory originates from evaluation research in the social sciences to explain how social interventions produce outcomes through explicit causal pathways [4]. This tradition later informed theory-driven evaluation approaches in health services research [6].

In this review, we understand a program broadly as any organized digital health initiative—ranging from single HIT implementations to complex, multicomponent interventions, such as disease management programs that include HIT as one component—that seeks to produce change at the individual, organizational, and/or system level.

Two prominent operationalizations of program theory are logic models and theory of change (ToC). Traditional logic models provide simplified, often linear depictions of inputs, activities, outputs, and outcomes to support planning, communication, and evaluation of complex interventions. However, they often underspecify mechanisms on how, why, and under which conditions elements are linked [5,7].

In contrast, ToC makes causal pathways explicit, visually mapping how and why an intervention leads to impact [7]. ToC thus places greater emphasis on causal links, context, and preconditions, articulating the evidence that underpins each link. Theories from sociology, behavioral science, or other disciplines can be inserted in the ToC to explain why specific links hold. Both logic models and ToC support planning, implementation, and evaluation, but they differ in flexibility, granularity, format, and how explicitly they depict causal pathways [7].

The UK Medical Research Council (MRC) has been central in mainstreaming logic models and program theory for complex health-related interventions. The 2015 MRC guidance on process evaluation formalized how to examine implementation, mechanisms, and context [2]. The 2021 MRC update reframed development and evaluation as iterative processes, positioning program theory and interaction between the intervention and its context as core elements to consider [3]. Integrating ToC into the MRC approach has been proposed to strengthen stakeholder engagement, articulate assumptions, and guide indicator selection along causal pathways [7].

Across disciplinary fields, the use of program theory is diverse. In public health, ToC and logic models have been used to structure multilevel, multifaceted, and multisectoral interventions [5]. In health technology assessment (HTA), logic models help identify how the complexity and context explain the outcomes of the interventions, both in single studies and in systematic reviews [8]. In implementation science, logic models have been used to support the choice of implementation strategy [9] and to build an implementation research logic model (IRLM) that links determinants, strategies, mechanisms, implementation outcomes, and clinical outcomes [10]. Methodological advances have led to typologies of logic models that explicitly account for the complexity that arises when interventions adapt to their context [11].

### Research Problem

Although HIT interventions are complex, the explicit use of program theory in medical informatics remains limited. Some examples illustrate their potential, including a ToC for electronic health record (EHR) implementation in Nigeria [12], a theory-informed internet intervention for emotional distress in primary care [13], a logic model for patient portals [14], or a ToC for extracting evidence from HIT studies [15]. Yet, these seem exceptions rather than standard practice in medical informatics.

At the same time, HIT is frequently embedded as one component within complex programs studied in public health, HTA, and implementation science where logic models and related approaches are routinely used. Examples include a maternal health program based on a digital exchange platform [16], an early intervention rehabilitation service for children integrating telerehabilitation [17], or an app-based intervention for cardiac care at home [18].

To our knowledge, no review has systematically examined how logic models and ToC are used in HIT-related interventions. Given their widespread use in other fields, clarifying their application in HIT is essential for theorization, evaluation, and cumulative learning in medical informatics. Mapping how logic models and ToC are conceptualized, constructed, and applied can reveal how HIT is framed, implemented, and evaluated, and generate methodological insights to strengthen medical informatics as a theory-informed discipline [19].

### Research Question

This scoping review aims to answer the following questions:

1. How are logic models for HIT-related interventions conceptualized, constructed, and applied in disciplines such as medical informatics, health services research, and health policy?
2. What practical consequences result from this for medical informatics research across clinical, public health, and policy settings?

## Methods

### Overview

The reporting of this review adheres to the PRISMA-ScR (Preferred Reporting Items for Systematic Reviews and Meta-Analyses extension for Scoping Reviews) [20]. The protocol was developed and published a priori [21].

### Eligibility Criteria

We included studies that met the following criteria (Population Concept Context [PCC] framework):

- Population: Studies and reports focusing on HIT interventions, including, but not restricted to, EHRs, clinical decision support systems, telemedicine platforms, mobile health (mHealth) apps, and patient portals.
- Concept: Explicit use or description of program theory approaches, including logic models, ToC, program logic, program theory, results chains, or related conceptual frameworks. Logic models may have been used for purposes such as design, implementation, evaluation, or communication.
- Context: Health care and social care settings, including hospitals, community care, long-term care, and underserved populations.
- Types of sources: Empirical studies (quantitative, qualitative, and mixed methods), conceptual or methodological papers, case studies, and evaluations.
- Language: English.
- Time frame: No restrictions on publication date.

We excluded studies presenting theoretical models that do not describe program theory or logic models in relation to a specific HIT. Papers, commentaries, editorials, or conference abstracts without sufficient detail were likewise excluded.

### Information Sources

A systematic search was done in PubMed, in Web of Science and (via EBSCOHost) in Academic Search Elite, APA PsycArticles, and CINAHL. Searches were complemented by AI-assisted search tools (Semantic Scholar, Connected Papers, ResearchRabbit, and Elicit).

### Search Strategy

The search strategy was developed iteratively, combining controlled vocabulary (MeSH) and free-text terms for four conceptual blocks:

1. Logic models and related terms (such as “logic model*,” “theory of change,” “results chain,” “programme logic,” and “program theory”).
2. HIT (such as “medical informatics,” “health information systems,” “digital health,” “electronic health record,” “electronic health record,” “telemedicine,” “clinical decision support systems,” and “mHealth”).
3. Application and purpose (such as “program evaluation,” “implementation science,” “evaluation,” “design,” “planning,” and “development”).
4. Care setting (such as “healthcare,” “social care,” “community care,” and “long-term care”).

The search query is documented in Multimedia Appendix 1.

### Screening and Data Extraction

Screening was conducted in 2 stages. First, the title and abstract of all records were screened by 2 independent reviewers (EA and IH) using the predefined inclusion and exclusion criteria. Discrepancies were resolved through discussion. Second, the full text of potentially eligible studies was screened, again by 2 independent reviewers (EA and IH). Reasons for exclusion at the full-text stage were recorded.

A structured data extraction form was developed, piloted, and iteratively refined. The form included the following domains:

- Bibliographic details (journal, year, and country).
- Study context (objective, health care setting, type of HIT system, and phase of HIT lifecycle).
- Characteristics of the logic model (purpose, novelty, level of system modeling, visual representation, components of the logic model, guidelines or frameworks guiding the development of the logic model, and frameworks or theories for populating the logic model).
- Usefulness (reported benefits and challenges).

Extraction was supported by AI-based tools (eg, ChatGPT [OpenAI]-based extraction). Each extraction was verified and revised manually by 2 reviewers (EA and IH) to ensure accuracy.

### Synthesis of Results

Our synthesis of results focuses on the health system levels addressed, the benefits and challenges explicated, and the theoretical foundations that the logic models are built on, in terms of the guidelines, frameworks, and theories used to structure the model and populate its content.

We used a combination of quantitative and qualitative synthesis approaches. Health care settings and HIT type were classified into categories. The phases of HIT interventions were classified based on the MRC 2021 framework into development, piloting (feasibility), evaluation, and scaling-up [3].

We categorized each logic model according to the health system level it addressed. The micro level referred to individuals and direct care processes (eg, patient-provider interactions). The meso level captured organizational or institutional dimensions (eg, workflows and management structures), while the macro level encompassed regional or national health system and policy contexts. Studies could address one or several levels simultaneously.

To allow for a more precise analysis of the theoretical foundations of logic models and theories of change, we distinguished a priori between two complementary types of theoretical guidance: (1) structuring guidance (used to build and organize the model) and (2) content-populating frameworks and theories (used to specify determinants, mechanisms, and context).

For structuring guidance, we extracted guidelines and frameworks on how to develop a logic model or a ToC, such as the MRC framework, Kellogg Logic Model [22], or IRLM [10]. These guidelines and frameworks provide overarching meta-methodological guidance on how to conceptualize and develop a logic model.

For content-populating frameworks and theories, we extracted frameworks or theories guiding the population of the model’s content, such as the theory of planned behavior [23] or the Consolidated Framework for Implementation Research (CFIR) [24]. These frameworks and theories inform the specific causal mechanisms, determinants, and contextual factors that are represented within the model. These frameworks and theories help to explain why and under what conditions a specific intervention is expected to produce its intended outcomes.

Reported benefits and challenges were analyzed using inductive quantitative text analysis.

No formal risk-of-bias or quality assessment was performed, consistent with scoping review methodology.

### Ethical Considerations

Ethics approval was not required as this review is based on secondary analysis of published literature.

## Results

### PRISMA Diagram

**Figure 1.**
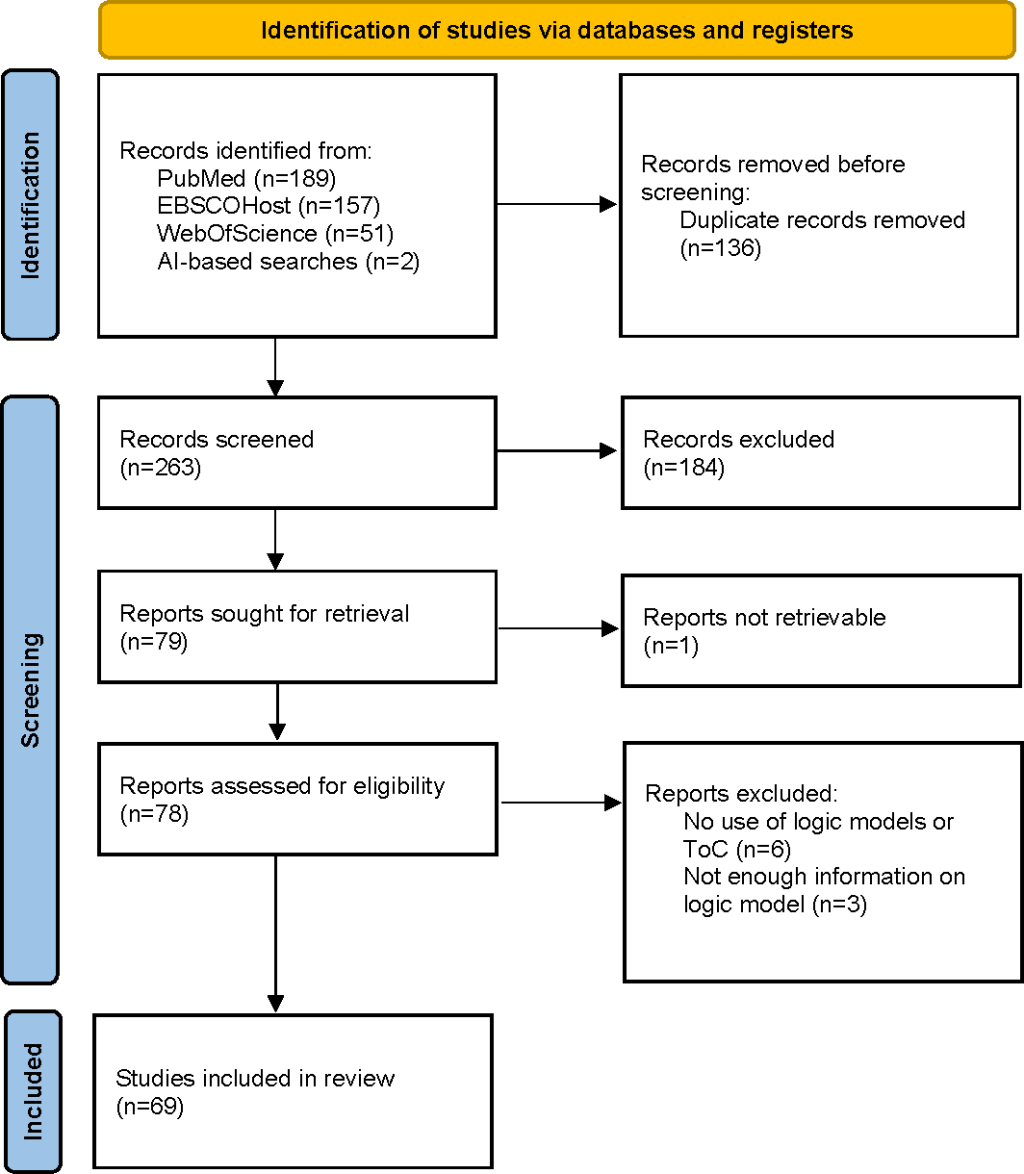
PRISMA (Preferred Reporting Items for Systematic Reviews and Meta-Analyses) statement flow diagram. ToC: theory of change.

The PubMed query retrieved 189 records. Combined with additional queries in other reference databases, the search yielded 263 unique records. AI tools contributed only 2 references. After checking inclusion and exclusion criteria, 69 papers [12,13,16-18,25-77] remained to be included in the review (Figure 1). Of the 69 studies, 58 (84%) papers [12,13,15-18,25-87] were primary studies, 6 (9%) papers were reviews [15,78-82], and 5 (7%) papers were study or review protocols [83-87].

The extracted data for all 69 studies can be found in Multimedia Appendix 2.

### Year of Publication

**Figure 2.**
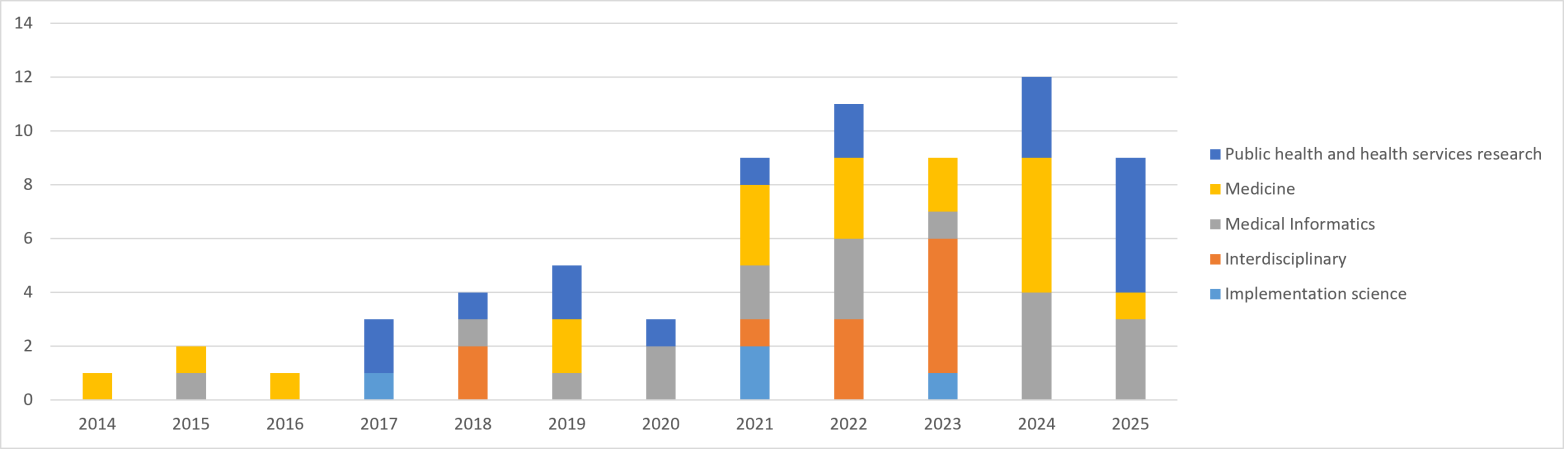
Publication year of the included studies (n=69 studies).

Included papers were published from 2012 to 2025, with a marked increase after 2020 (Figure 2).

Out of 69, 19 (27%) papers were published in medical journals (including 1 nursing journal), 18 (26%) papers were published in journals that can be related to medical informatics as a discipline, and 17 (25%) papers were published in public health or health services research journals. Another 11 (16%) studies were published in interdisciplinary journals, and 4 (6%) papers in implementation science journals.

The clear post-2020 increase indicates a rapidly growing uptake of program theory in HIT, especially in early lifecycle phases. This variety indicates a broad interest from various fields in developing and evaluating HIT as part of health-related interventions.

### Country

**Figure 3.**
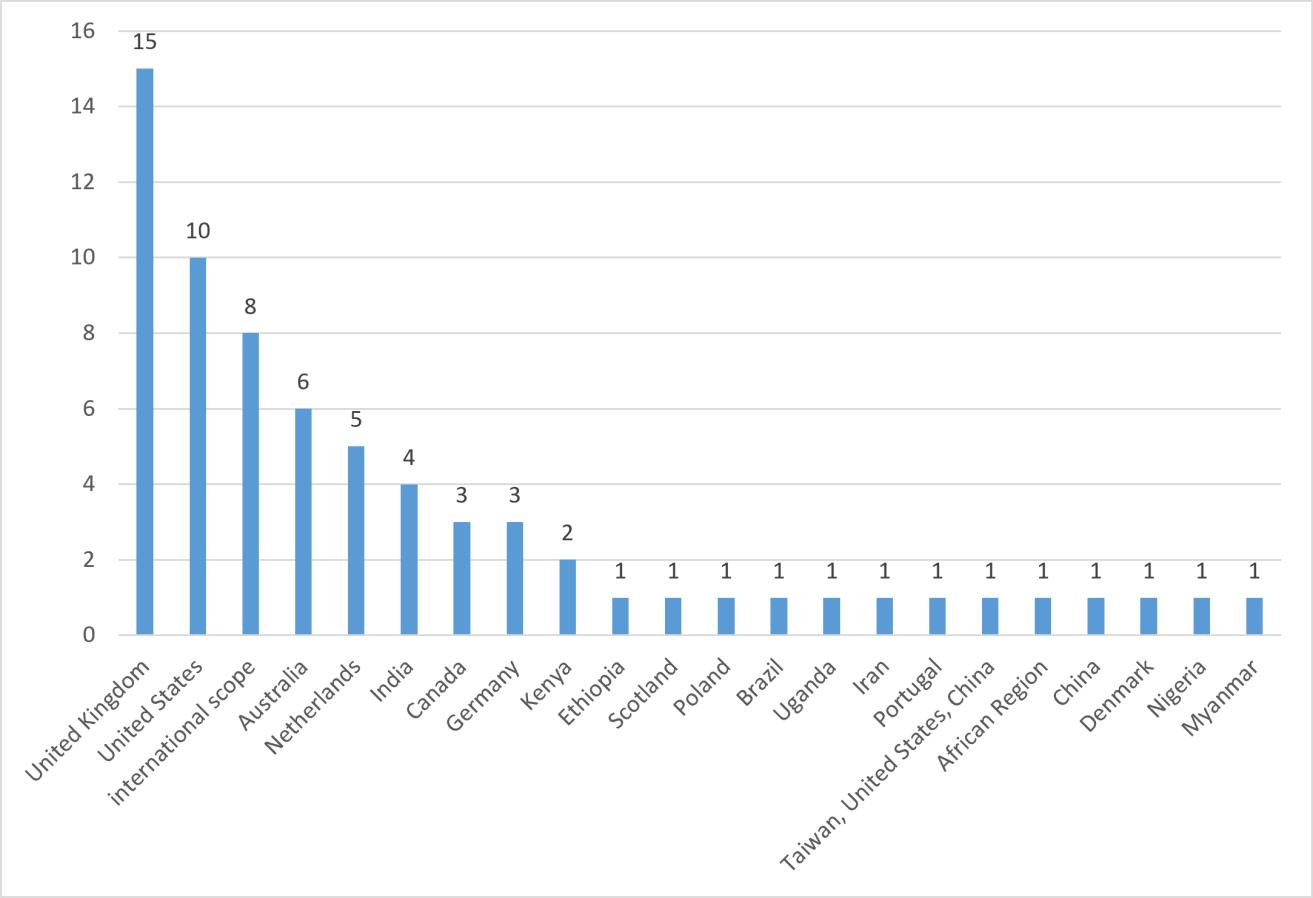
Country where a study took place or region covered by a review (n=69 studies).

Just over half of the included papers (n=36; 52%) came from just 4 countries: the United Kingdom, the United States, the Netherlands, and Australia. Moreover, 13 (19%) papers came from low-income countries such as India and Kenya (Figure 3).

Studies are therefore concentrated in a few high-income countries alongside notable contributions from resource-constrained settings.

### HIT and Health Care Setting

**Figure 4.**
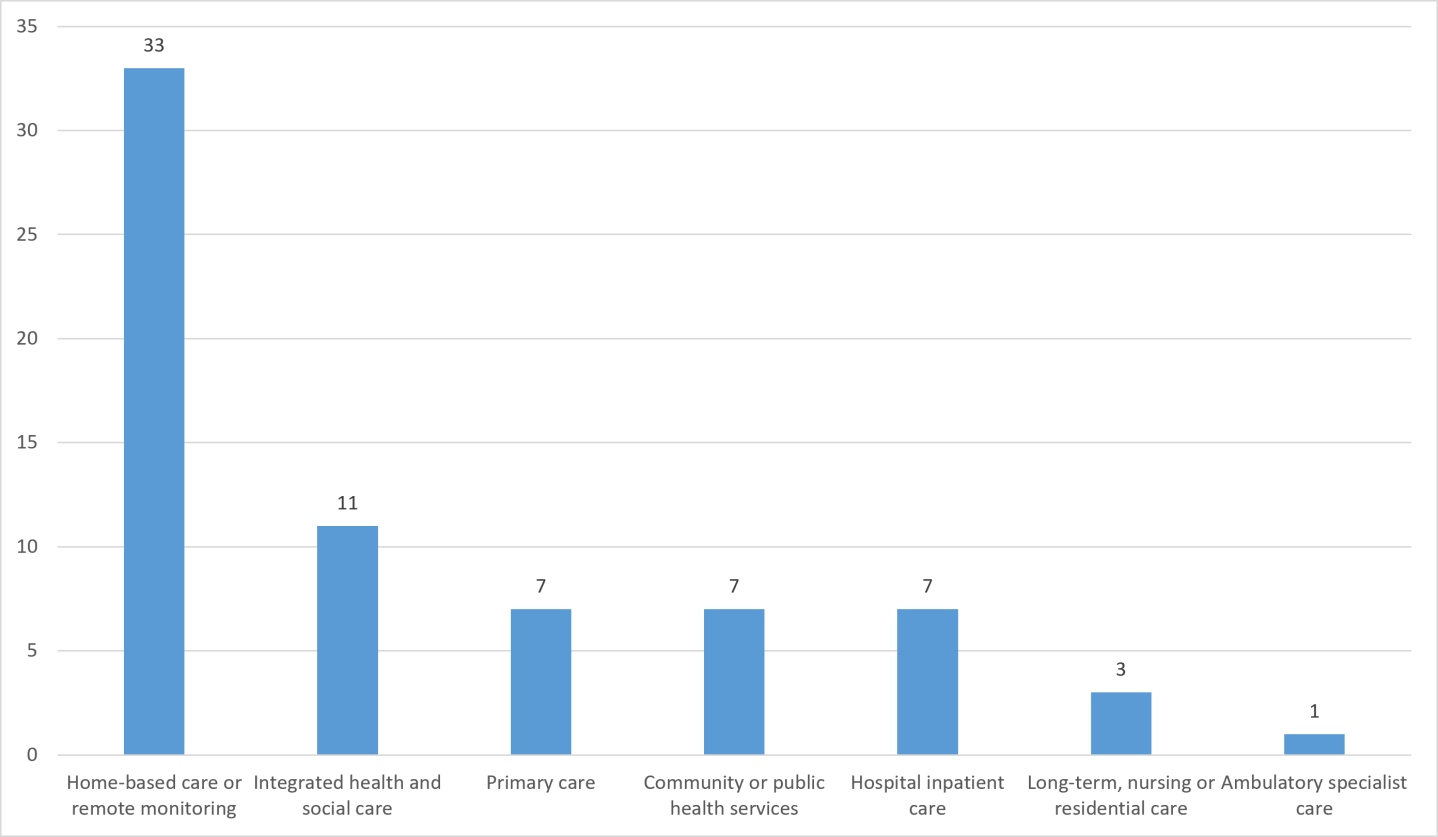
Health care setting where a study took place (n=69 studies).

About half of the studies (n=33, 48%) took place in home-based or remote health care settings where HIT is to be used by patients or their families. Examples are HIT for self-management of diabetes [46] or telemedical care of infants at home [40]. Moreover, 11 (16%) studies took place in integrated health and social care settings, which comprise interventions not limited to a specific health care setting but covering several settings, such as hospital and social care. Examples include a cross-sectoral intervention to improve youth mental health services [34]. The other studies were located in a variety of clinical settings such as hospitals or primary care (Figure 4).

**Figure 5.**
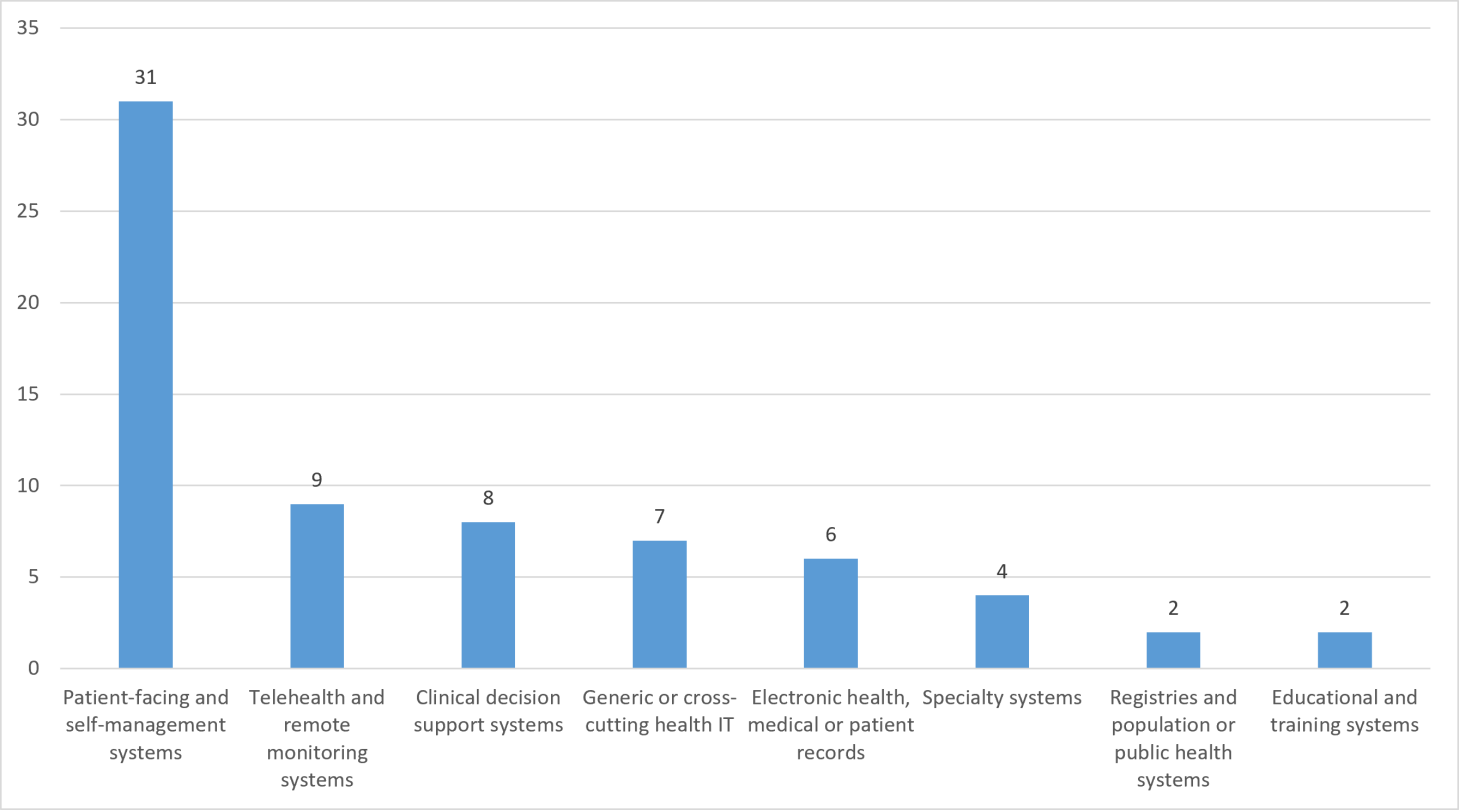
HIT type of included studies (n=69 studies).

About half of the studies (n=31, 45%) cover patient-facing and self-management applications, such as mHealth apps. The remaining studies (n=38) cover a mix of HIT types (Figure 5).

**Figure 6.**
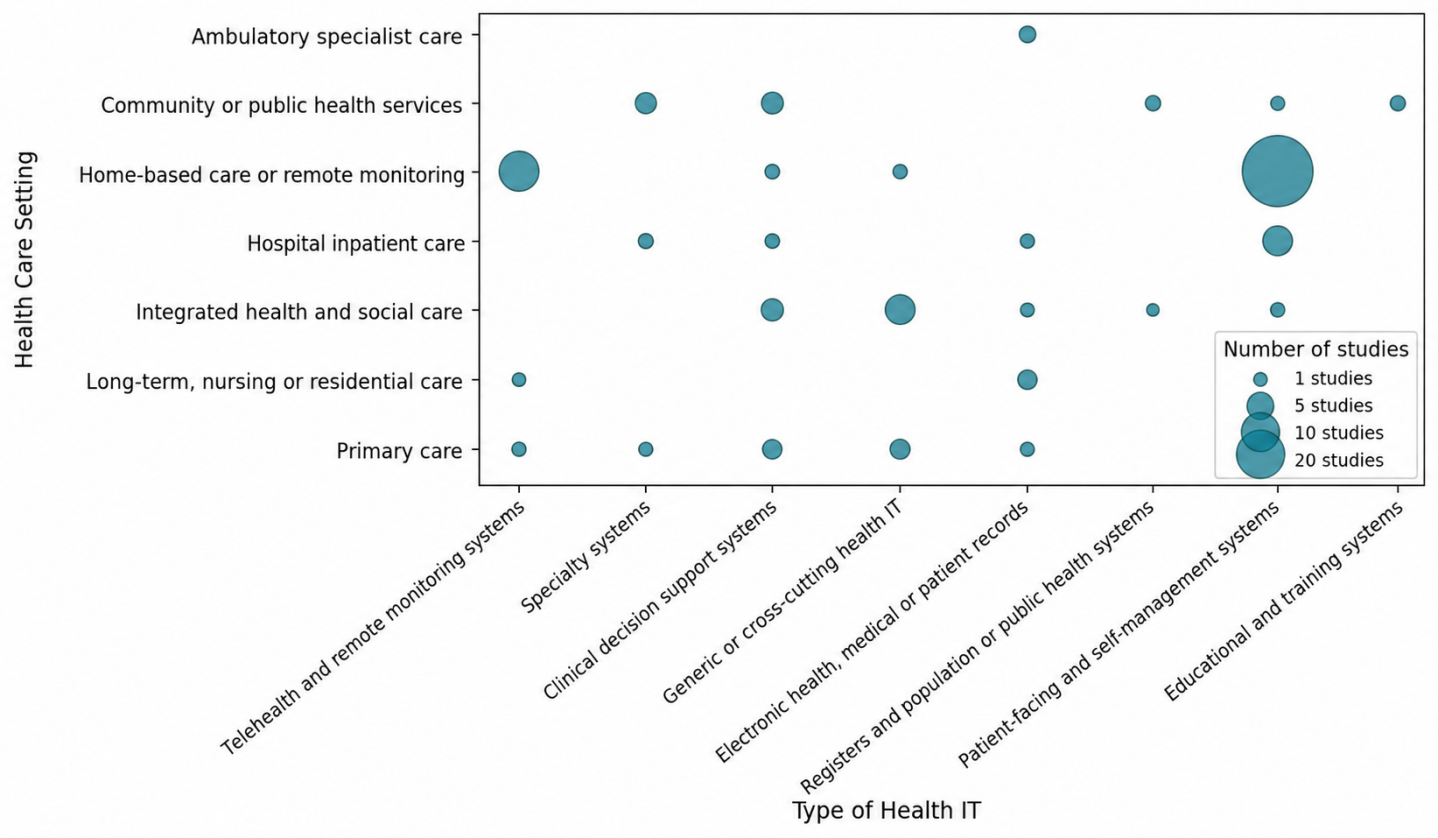
Relationship between type of Health Information Technology and health care setting (n=69 studies). The bubble size represents the number of studies using a logic model for each combination.

Figure 6 illustrates the correlation of logic model applications across different types of HIT and different care settings. Most studies focused on patient-facing and self-management systems, particularly in home-based or remote-care contexts. In contrast, EHR and clinical decision support systems were mainly represented in institutional settings, such as hospitals or primary care.

Summarizing, logic model use is most prevalent for patient-facing, home-based, and remote interventions, aligning with the need to capture behavioral and contextual mechanisms in these settings. Institutional HIT (eg, EHR or clinical decision support systems) is comparatively less represented and more often situated in the clinical environment.

### Level of System Modeling

Logic models and ToC were categorized to address the micro level of the health system, focusing on individuals and direct care processes; the meso level, focusing on the health care institution; and/or the macro level, thus focusing on the regional or national health system. Papers could address more than one level.

**Figure 7.**
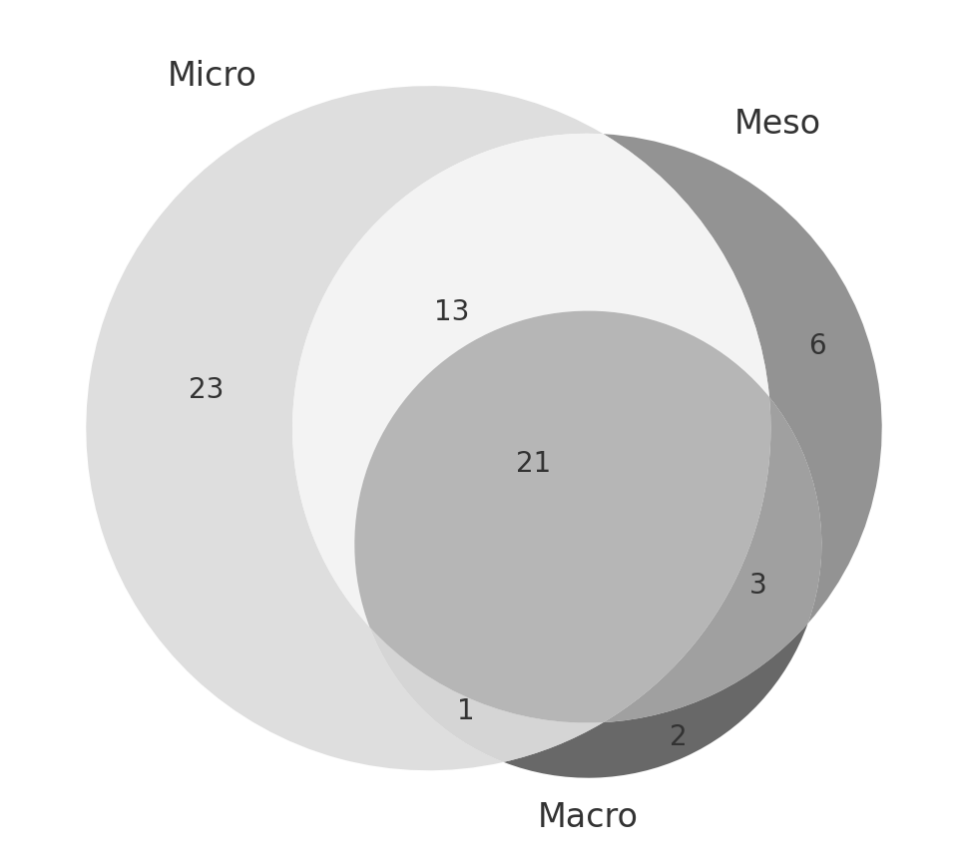
Overlap between micro, meso, and macro focus in the included studies (n=69).

Overall, 58 (84%) papers addressed the micro level, 43 (62%) papers addressed the meso level, and 27 (39%) papers addressed the macro level, with most papers addressing more than one level (Figure 7). For example, on the micro level, 1 study used a logic model to analyze how software features influence outcomes when developing a self-help digital intervention for young people with Tourette syndrome [28]. On the meso level, 1 study developed a logic model to understand context factors relevant for adopting electronic medication administration systems in long-term care facilities [47]. On the macro level, 1 study developed a logic model to understand scaling-up of an electronic consult service across Canadian provinces [86].

In total, 21 studies covered all 3 levels (micro, meso, and macro). For example, 1 study investigated how context and mechanisms influence the outcomes of integrated care for type 2 diabetes in 2 Dutch care groups [32]. This study found barriers and facilitators on the individual patient and professional (micro) level (such as user resistance), on the organizational (meso) level (such as lack of qualified nurses), and on the political (macro) level (such as financial pressure and health insurer cooperation).

Summarizing, most models prioritize microlevel processes, with fewer addressing organizational and policy levels, indicating an emphasis on proximal change over systemic effects.

### Visualizations of Logic Models

A clear majority of the studies (60/69, 87%) presented a visualization of the logic model or referred to a visualization reported elsewhere. From these 60 studies, 6 studies applied the most condensed structure, the context-mechanism-outcome (CMO) configuration. These studies referred to realist evaluation as the theoretical foundation guiding the construction of the logic model. Most of the papers used more detailed structures; however, we could not identify a specific pattern suggesting that the theoretical guideline or framework chosen to guide the development of a logic model would define the components of the graphical representation.

All logic models with visualizations (n=60) included outcomes in their models. About half of these studies (31/60, 52%) categorized these outcomes based on their temporal distality (short-term or long-term outcomes) or their attributability to the intervention. About half of these studies (31/60, 52%) included context and/or determinants (also named “assumptions” and “enablers and barriers”) in the visualizations. A third of these papers (20/60, 33%) included the problem to be solved with the intervention (or “purpose” or “goal”) in the visualization.

Overall, the visualizations varied notably in how the intervention and its mechanisms were labeled, including labels such as inputs, technology, activities, intervention, mechanisms, processes, and implementation strategy.

### Purpose of Using Logic Models

Using the MRC 2021 framework [3], we analyzed which of the 4 phases of an intervention was supported by the logic model:

1. HIT development (n=39 studies, 57%): Logic models were used to support needs analysis, develop a program theory, identify components and mechanisms of the HIT intervention to be developed, and engage with stakeholders.
2. HIT piloting (n=20, 29%): Logic models were used for testing of acceptability, usability, and feasibility, for training, for small pilots, and for refining the program theory of the newly developed HIT intervention.
3. HIT evaluation (n=35, 51%): Logic models were used to support assessment of implementation fidelity, economic aspects, and effectiveness of the HIT intervention.
4. HIT scaling-up (n=11, 16%): Logic models were used to support sustainability of the HIT intervention in real-world contexts, to scale it up to other settings, and to evaluate long-term effects.

Most papers covered more than one phase of the intervention phases. For example, 12 studies covered development and piloting; 5 studies covered evaluation and implementation; and another 5 studies covered development, piloting, and evaluation.

**Figure 8.**
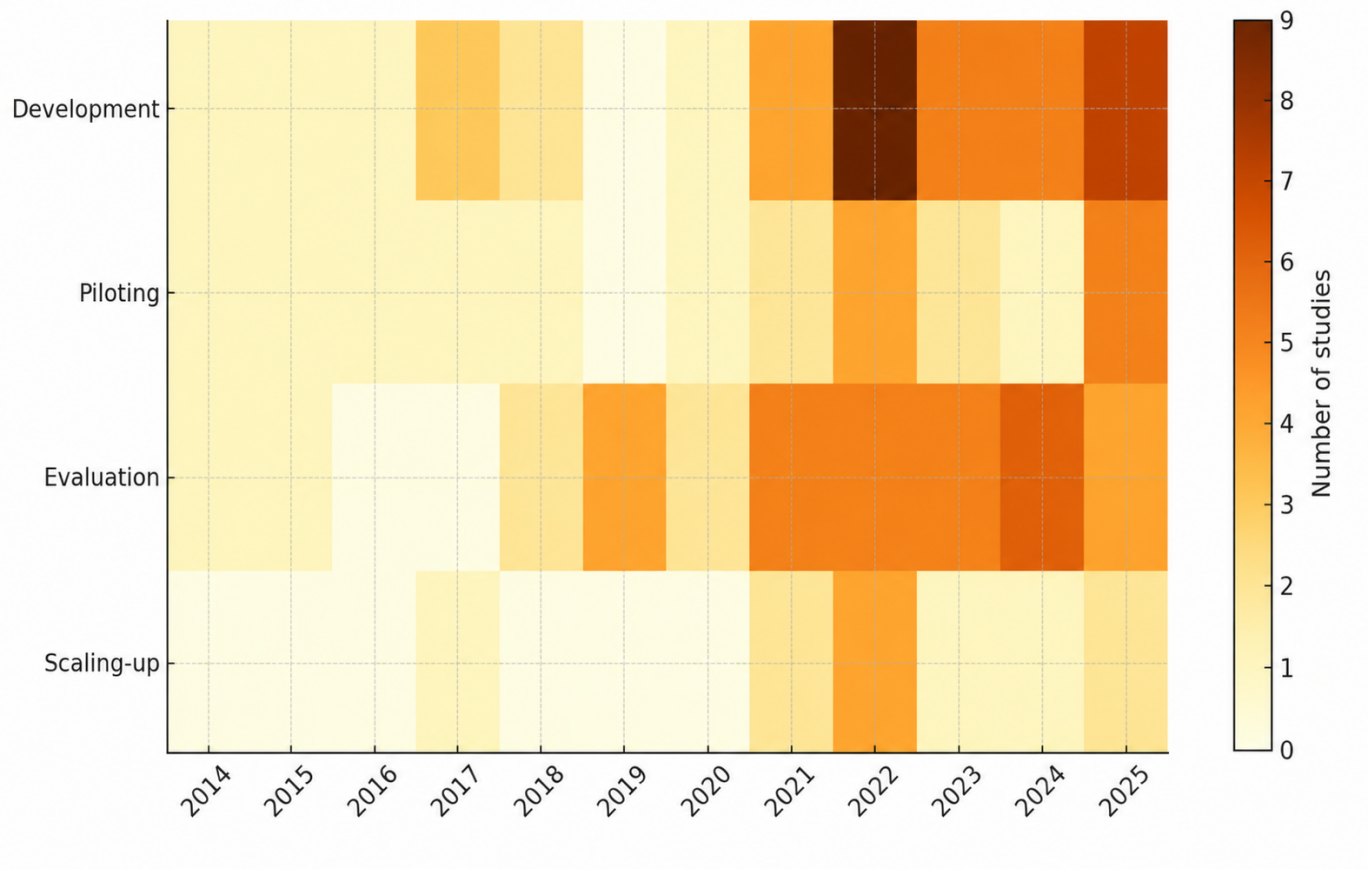
Use of logic models across different phases of the health information technology lifecycle over time (2014‐2025).

To illustrate the temporal distribution of logic model applications, all 69 included studies were analyzed by publication year and lifecycle phase (development, piloting, evaluation, and scaling-up). Figure 8 displays aggregated frequencies per year and phase. Higher color intensity represents a larger number of studies. The use of logic models across all lifecycles phases increased strongly after 2020, with the development phase most dominant in all years.

Summarizing, logic models predominantly support development and evaluation, with fewer applications in piloting and scaling-up.

### Use of Existing Logic Models

Only 3 (4%) studies explicitly stated that they reused an existing logic model or ToC:

The first study developed a ToC for a new moderated online social therapy for youths. For building their ToC, the authors reviewed examples of ToC frameworks in comparable youth web-based mental health programs [34].

The second study developed a logic model to determine how eHealth was adopted in pharmaceutical care during COVID-19. For building this logic model, the authors reused a logic model for pharmaceutical care published by another group [79].

The third study presented a ToC describing how geospatial data and technologies can improve immunization coverage and equity. For building this ToC, the authors reused a ToC for the use of geospatial technologies for immunization programming published earlier [33].

The remaining studies either developed a new logic model or ToC (n=63, 91%) or did not give clear information on reusing an existing logic model (n=3, 4%). However, many studies referred to guidelines and frameworks that helped them to develop their logic model by providing templates or structural patterns – this is further analyzed in the next section.

### Guidelines, Frameworks, and Theories

As described in the Methods section, we differentiate between two types of theoretical guidance observed in the included studies: (1) guidelines or frameworks on how to develop a logic model or a ToC, and (2) frameworks or theories guiding the population of the model’s content.

### Guidelines or Frameworks on How to Develop a Logic Model

Overall, 50 (72%) studies presented information on which guidelines or frameworks on how to develop a logic model the studies used. Of these studies, 37 used 1 guideline or framework and 13 studies used 2 guidelines or frameworks. Out of 69, 19 (28%) studies did not mention any guideline or framework.

**Figure 9.**
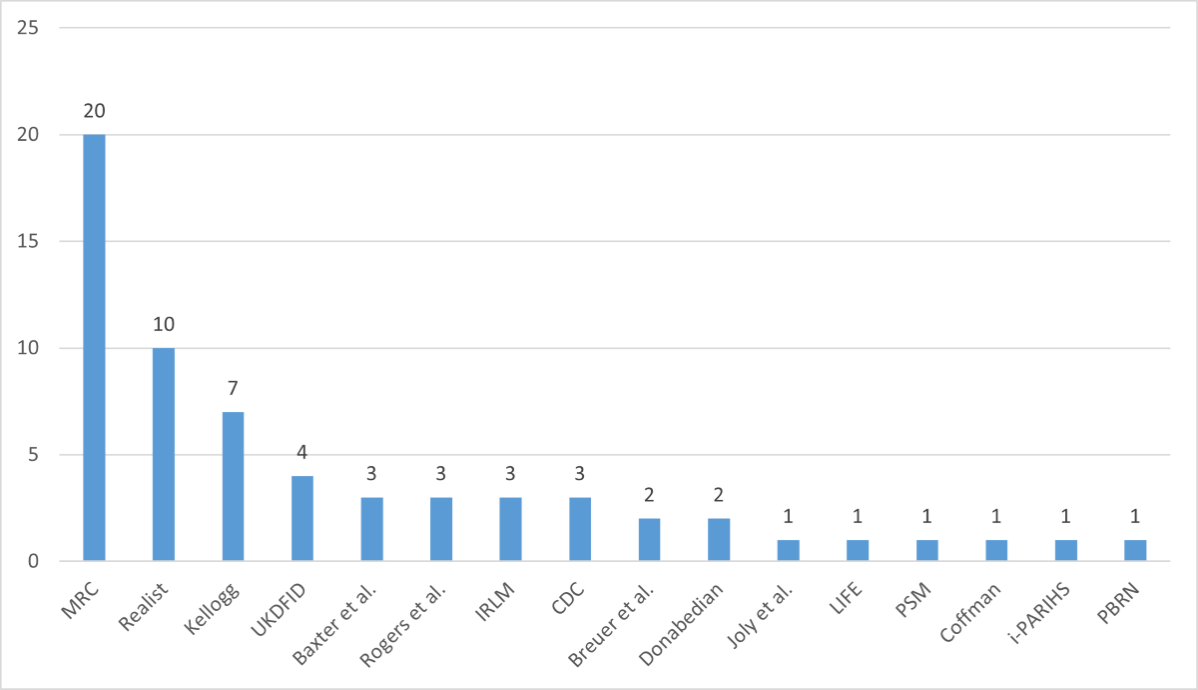
Guidelines and frameworks on how to develop a logic model as mentioned in 50 studies. For an explanation of all theories and frameworks, refer to Multimedia Appendix 3. CDC: Centers for Disease Control and Prevention; i-PARIHS: Integrated Promoting Action on Research Implementation in Health Services; IRLM: Implementation Research Logic Model; LIFE: Local Initiatives for Faith and Evaluation; MRC: Medical Research Council guidance; PBRN: Practice-Based Research Network; PSM: Participatory Systems Mapping; UKDFID: UK Department for International Development.

Figure 9 illustrates the guidelines and frameworks used to develop the logic model by the 50 studies. None of the identified guidelines and frameworks is specific for HIT or health informatics.

The most frequently mentioned framework is the MRC guidance (20 studies, 29% of all studies), which provides a comprehensive framework for developing and evaluating complex interventions in health and social care [3]. The MRC guidelines have been updated frequently and are often cited as meta-guidance for developing and structuring logic models.

The next most frequent category is “Realist Evaluation” (10 studies, 15%), indicating that the authors seek to explain how, why, for whom, and under what conditions interventions work by analyzing the interaction of context, mechanisms, and outcomes (CMO configurations).

The third most often mentioned framework is the Logic Model Development Guide of the Kellogg Foundation (7 studies, 10%), which is a practical manual that explains how to design, apply, and interpret logic models for program planning and evaluation [22]. It is one of the oldest, most widely used nondisciplinary guides for constructing program theory.

For explanation and references for each guideline and framework, refer to Multimedia Appendix 3.

**Figure 10.**
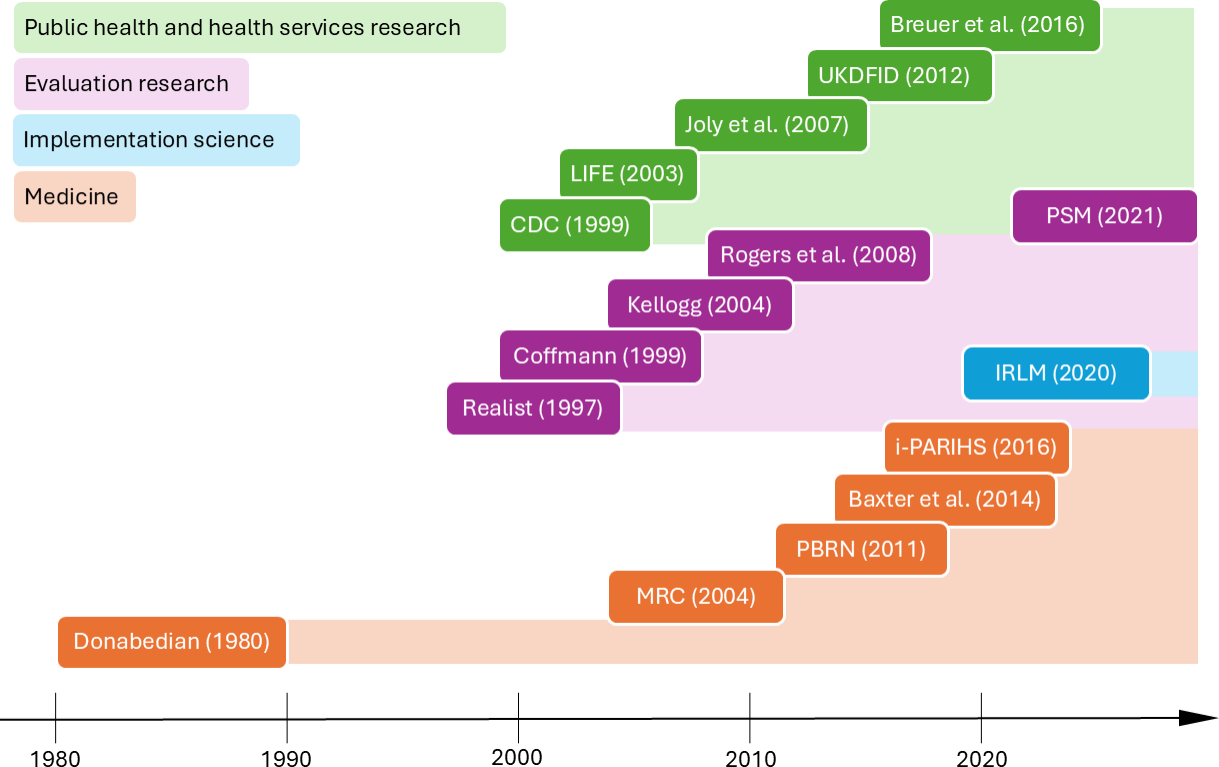
Timeline of guidelines and frameworks used to develop logic models in 50 studies. The x-axis shows the publication year of the study that cited each guideline or framework. For an explanation of all theories and frameworks, refer to Multimedia Appendix 3. CDC: Centers for Disease Control and Prevention; i-PARIHS: Integrated Promoting Action on Research Implementation in Health Services; IRLM: Implementation Research Logic Model; LIFE: Local Initiatives for Faith and Evaluation; MRC: Medical Research Council guidance; PBRN: Practice-Based Research Network; PSM: Participatory Systems Mapping; UKDFID: UK Department for International Development.

Figure 10 illustrates the timeline of these guidelines and frameworks used to develop a logic model. We mapped their chronological appearance across disciplinary domains based on the versions referenced in the included papers. The assigned domains are indicative; several guidelines and frameworks could be appropriately classified into more than 1 domain.

Summarizing, most studies cite structuring guidance, led by MRC, realist evaluation, and Kellogg, none of which are HIT-specific.

### Frameworks and Theories for Populating the Logic Model

Overall, 28 (41%) studies presented information on which frameworks and theories they used to populate their logic model. Out of 69, 15 (22%) studies used 1 framework or theory, 4 (6%) studies used 2, 8 (12%) studies used 3, and 1 (1%) study even used 4 frameworks or theories. Moreover, 41 (59%) studies did not mention any framework or theory.

**Figure 11.**
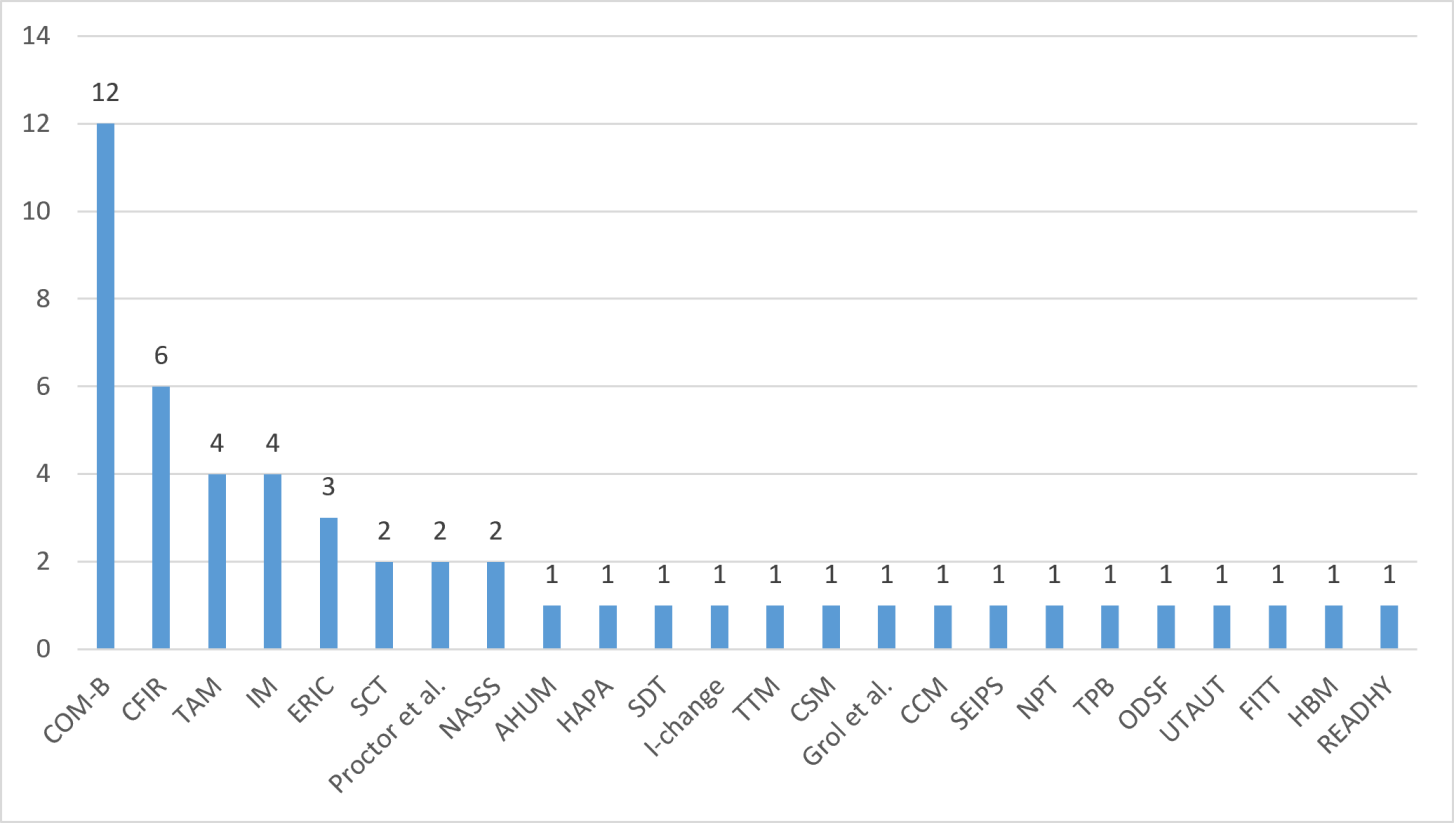
Frameworks and theories for populating a logic model as mentioned in 28 studies. For an explanation of all frameworks and theories, refer to Multimedia Appendix 3. AHUM: Andersen Healthcare Utilization Model; CCM: Chronic Care Model; CFIR: Consolidated Framework for Implementation Research; COM-B: Capability, Opportunity, Motivation–Behavior; ERIC: Expert Recommendations for Implementing Change; FITT: Individual, Technology and Task Framework; HAPA: Health Action Process Approach; HBM: Health Belief Model; HIT: Health Information Technology; IM: Implementation Mapping; NASSS: Nonadoption, Abandonment, Scale-up, Spread, and Sustainability; NPT: Normalization Process Theory; ODSF: Ottawa Decision Support Framework; READHY: Readiness and Enablement Index for Health Technology; SCT: social cognitive theory; SDT: self-determination theory; SEIPS: Systems Engineering Initiative for Patient Safety; TAM: technology acceptance model; TTM: Transtheoretical Model of Health Behavior Change; UTAUT: unified theory of acceptance and use of technology.

Figure 11 illustrates the frameworks and theories used by the 28 studies. Only the Fit between Individuals, Task, and Technology (FITT) framework [88] and the Non-adoption, Abandonment, and challenges to the Scale-up, Spread, and Sustainability (NASSS) framework [89] originated in health informatics; the other frameworks and theories are rooted in other domains.

The most frequently mentioned framework (11 studies, 16% of all studies) is the Capability, Opportunity, Motivation–Behavior model (COM-B), which identifies that behavior results come from addressing capability, opportunity, and motivation. COM-B is the core of the behavior change wheel framework [90].

The second most frequently used framework is the CFIR (6 studies, 10%), which is a theory-based framework used to guide the implementation of complex interventions in health care and related fields [24]. CFIR describes determinants of implementation success.

For explanation and references for each found framework or theory, refer to Multimedia Appendix 3.

**Figure 12.**
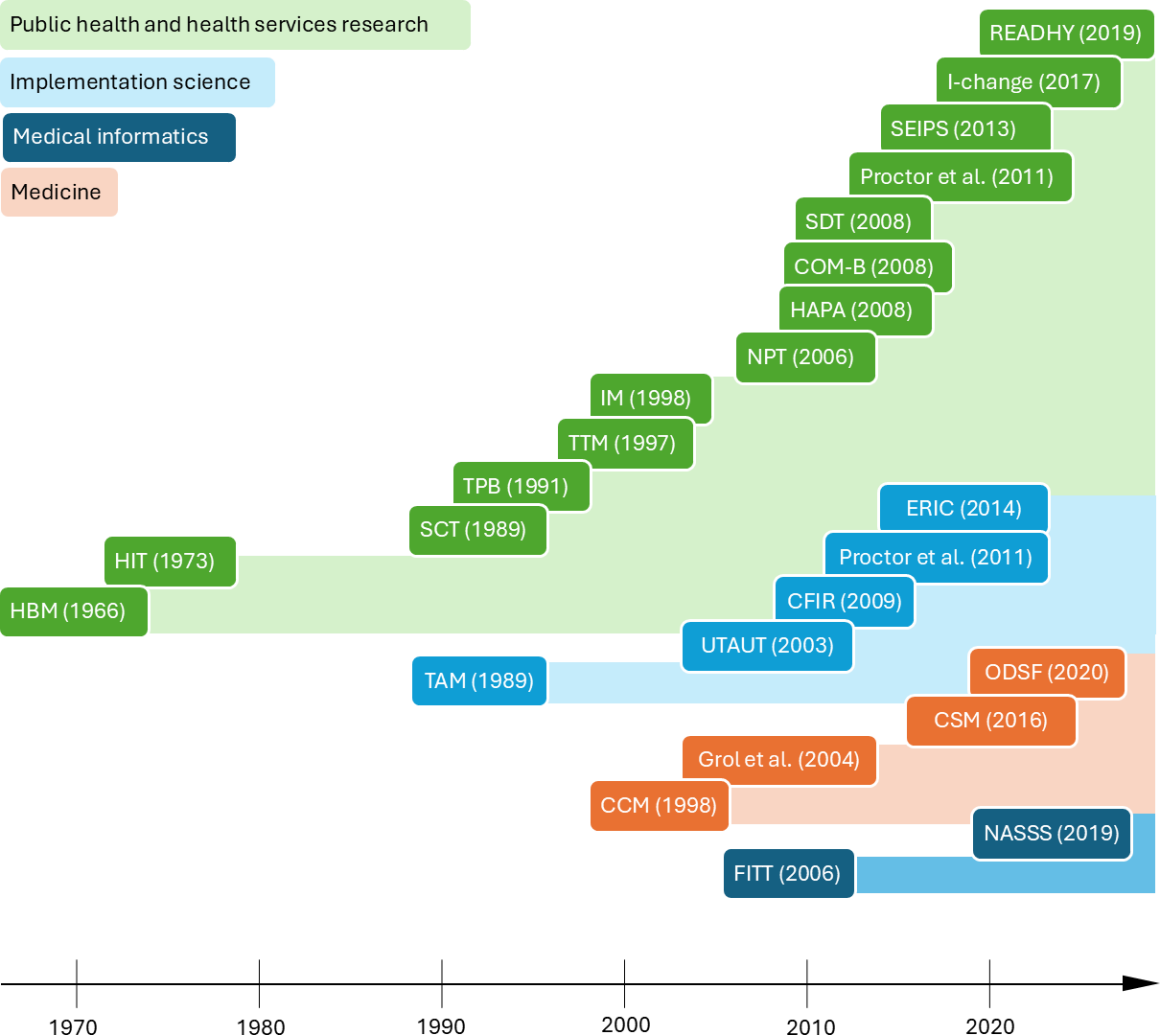
Timeline of frameworks and theories for populating a logic model as used in 28 studies. The x-axis shows the publication year of the study that cited each framework or theory. For an explanation of all theories and frameworks, refer to Multimedia Appendix 3. CCM: Chronic Care Model; CFIR: Consolidated Framework for Implementation Research; COM-B: Capability, Opportunity, Motivation–Behavior; ERIC: Expert Recommendations for Implementing Change; FITT: Individual, Technology and Task Framework; HAPA: Health Action Process Approach; HBM: Health Belief Model; HIT: Health Information Technology; IM: Implementation Mapping; NASSS: Nonadoption, Abandonment, Scale-up, Spread, and Sustainability; NPT: Normalization Process Theory; ODSF: Ottawa Decision Support Framework; READHY: Readiness and Enablement Index for Health Technology; SCT: social cognitive theory; SDT: self-determination theory; SEIPS: Systems Engineering Initiative for Patient Safety; TAM: technology acceptance model; TTM: Transtheoretical Model of Health Behavior Change; UTAUT: unified theory of acceptance and use of technology.

Figure 12 illustrates the timeline of these frameworks and guidelines. We mapped their chronological appearance across disciplinary domains based on the versions referenced in the included papers. The assigned domains are indicative; several frameworks and theories could be appropriately classified into more than 1 domain.

Summarizing, logic model content is commonly populated with behavioral and implementation frameworks (notably COM-B and CFIR), with only NASSS and FITT originating in health informatics.

### Model Components

Some logic models provided clear causal linkages between inputs, activities, outputs, and outcomes, while others were schematic or purely illustrative, lacking operational definitions or indicators.

For example, 1 study presented a logic model of around 10 items organized into CMO to describe the uptake of a vision screening program [85,91]. Another study presented a logic model of around 40 items, organized into problems, inputs, intervention, outputs, measurable effects, wider benefits, and long-term change to describe the introduction of EHRs for maternal and child health [12]. This shows the varying level of details among the included studies.

### Reported Benefits and Challenges of Using Logic Models

Overall, we could extract 92 statements on perceived benefits of using logic models from 51 papers. The remaining 18 papers did not report any specific benefits of using a logic model. Reported benefits focus around clarifying causal assumptions, supporting targeted implementation and evaluation, and enabling stakeholder dialogue (Table 1).

**Table 1.** Reported benefits of using a logic model (from n=51 studies).

Reported benefits of using logic models	Studies, n
Support targeted, theory-based implementation	21
Explicate how the intervention leads to outcomes and long-term change	13
Identify success factors and potential causes of problems	13
Support testing and evaluation	12
Synthesize evidence	9
Support dialogue with stakeholders	9
Show areas for improvement	6
Articulate and clarify assumptions	4
Allow generalization to other contexts	3
Captures learning for future implementations	2
Sum	92

Also, we could extract 58 statements on perceived challenges of using logic models from 40 papers. The remaining 29 papers did not report any specific challenges of using a logic model. Challenges include limited evidence to populate models, resource intensity, and tensions between specificity and generalizability (Table 2).

**Table 2.** Reported challenges of using logic models (from n=40 studies).

Reported challenges of using logic models	Studies, n
Not enough evidence available to build LM^a^	8
ToC^b^ is too general, not context-specific	7
ToC is too context-specific, cannot be generalized	7
Building ToC is time-consuming	7
Not all stakeholders were involved in LM development	6
Not all assumptions, factors, or outcomes can be measured	6
ToC does not cover full details	5
Difficult to choose the right LM template and tool	3
LM predictions were not met by reality	2
LM should include also practical evidence	2
Stakeholders may disagree	1
Stakeholder not trained in ToC	1
Social desirability reflected in ToC	1
Implementation was not fully done according to LM	1
Difficult to select the right theory	1
Total replies	58

a LM: logic model.

b ToC: theory of change.

## Discussion

### Principal Results

This scoping review identified 69 studies that developed or applied a logic model or ToC in the context of HIT. The number of publications has increased markedly since 2020, suggesting a growing recognition of the importance of program theory approaches in general for conceptualizing and evaluating complex HIT interventions.

Across the included studies, the most frequent purposes of using logic models were descriptive (to explain program components and mechanisms) and evaluative (to structure data collection and outcome assessment). Most logic models were newly developed for a specific project. Yet, they were often built on existing frameworks or theories that provide templates or guidance to build logic models for a specific domain or context (such as IRLM).

Geographically, while most studies originated from high-income countries, a considerable share came from resource-constrained settings. Many of these studies focused on HIT designed to improve health care access or to support service coordination, particularly mobile and telehealth interventions. Frequent use in resource-constrained contexts partly reflects funder requirements that encourage ToC or logic models for planning, monitoring, and evaluation [92,93]. Thus, their relatively high representation may reflect reporting practices within international development programs.

Regarding the types of technologies, logic models were most often applied to patient-facing and self-management systems (eg, mHealth apps) and to interventions involving telehealth and remote monitoring. In contrast, institutional HITs, such as EHR, were less frequently represented. This may reflect greater user interaction, context dependence, and behavior change in patient-facing technologies; areas where program theory helps capture mechanisms.

In our review, a broad spectrum of theories and frameworks was identified; yet, their application clustered around a few recurrent approaches. Frameworks such as the MRC guidance, Kellogg, or IRLM were often used as a guide on how to develop a logic model, while behavioral models and implementation-oriented frameworks, such as COM-B, CFIR, NASSS, FITT, or Unified Theory of Acceptance and Use of Technology (UTAUT), often informed the population of logic model content. This dual pattern reflects a distinction—methodological frameworks provide structure, while theoretical frameworks explain behavioral and contextual mechanisms.

Many studies combined elements from multiple frameworks or theories when populating their logic model, resulting in considerable conceptual heterogeneity of the developed logic models. An earlier review of HIT evaluation frameworks identified 6 dominant frameworks (CFIR; Reach, Effectiveness, Adoption, Implementation, and Maintenance Framework; technology acceptance model; UTAUT; diffusion of innovation theory; and normalization process theory) which guided HIT evaluation [91]. As we covered more phases besides HIT evaluation, we found a much greater heterogeneity of frameworks and theories.

The visualization of logic models was common, as almost all included studies presented at least 1 graphical representation. However, the level of structure and detail of these visualizations varied. Most visualizations lacked visible alignment with a guidance or framework such as the MRC or Kellogg. This suggests that model structure and graphics are driven more by practical needs and study phase than by a specific theoretical framework. This contextuality may limit comparability and reuse of logic models across studies.

This diversity in the included logic models may thus be related to both the type of HIT and the methodological purpose or phase of use. Logic models describing patient-facing or behavioral interventions, such as mHealth apps or telemonitoring systems, often adopted detailed, multilayered visualizations to represent behavioral mechanisms and contextual enablers. In contrast, logic models for institutional HIT, such as EHRs or decision support tools, tended to remain schematic, focusing on input-process-output sequences that mirror technical implementation steps rather than theoretical constructs.

The analysis by system level revealed that logic models were predominantly situated at the micro level, focusing on individual users, care teams, or patient-provider interactions. Fewer studies addressed the meso level (organizational or workflow integration), and even fewer considered the macro level (policy or system-wide implementations). This pattern shows that logic models in HIT focus more on project-level use than on system or policy assessment. Considering the systemic nature of many HIT solutions, including macro level considerations is recommended in future studies.

Several benefits of logic model use were reported across studies. Logic models helped clarify intervention components and causal assumptions, facilitated stakeholder engagement, and supported alignment between program design and evaluation. They also served as communication tools among interdisciplinary teams and as frameworks for monitoring the intervention over time. Some studies emphasized that the process of developing a logic model – rather than the final visualization – was most valuable for building shared understanding and identifying priorities.

Conversely, the main found challenges included lack of evidence to populate the logic model, the resources required for model development, and the trade-off between specificity and generalizability of the logic model. Earlier studies had also noticed that resource management may be a critical issue, especially in staged processes of logic model development where the model is iteratively developed together with multiple stakeholder groups [94].

### Trade-Offs of Logic Models for HIT

Our findings highlight a core tension—logic models help structure complexity; yet, their use requires balancing complexity versus feasibility and specificity versus generalizability.

The first key trade-off concerns the complexity versus feasibility. Highly detailed logic models can capture the multifaceted interactions between technology, users, and context, but they risk becoming complex [94] and difficult to operationalize in real-world settings. Conversely, simpler models are easier to communicate but may obscure critical mechanisms and contextual dependencies. This prompts the question of what constitutes a “good” logic model that can balance simplicity, comprehensiveness, and interpretability. Previous work has shown that visual models play a key role in communicating complex information system structures and their effects [95]. Yet, there seems to be no shared benchmarks for an optimal level of abstraction for HIT logic models in different settings.

A second trade-off concerns the balance between specificity and generalizability. Logic models developed for narrowly defined interventions (such as mHealth or telemonitoring programs in maternal care) offer detailed causal mapping but have limited transferability beyond their original context. Interventions depicted in a narrow manner remain difficult to compare with other interventions, and to reproduce and apply in other settings. In contrast, models grounded in general frameworks (eg, MRC and Kellogg) enable cross-study comparison but often require substantial adaptation to each setting. Better awareness of general frameworks among local researchers and developers could encourage reporting of balanced logic models with sufficient generalizability and specificity.

In our review, only 3 studies explicitly reported building their model on an earlier one, highlighting that reuse of existing logic models remains rare. Accessible repositories, combined with domain-specific frameworks, could help researchers build on previous models and improve methodological continuity. This could address the current fragmentation that may contribute to a replication crisis in health informatics [96].

### Practical Steps to Enable Reuse

To translate the goal of reuse into practice, we suggest a minimal set of reporting elements and infrastructure supports for HIT-related logic models; authors should provide structured, consistent metadata for each model: (1) intended purpose and lifecycle phases addressed (development, piloting, evaluation, and scaling-up); (2) health system level modeled (micro, meso, and macro); (3) guidelines used to develop the model (eg, MRC and Kellogg); and (4) frameworks or theories used to populate causal content (eg, COM-B, CFIR, and NASSS or FITT), together with a clear statement on whether the model is new, adapted, or reused from previous work. Standardized visualizations should explicitly depict components, outcomes over time, and contextual determinants and assumptions to aid comparability across studies. Providing these details consistently would reduce heterogeneity, enhance interpretability, and make logic models more readily adaptable across HIT settings.

Journals and funders could also require explicit program theory reporting for HIT (analogous to existing evaluation reporting standards such as Standards for Reporting Health Informatics [STARE-HI] [97]), and support the creation and use of accessible repositories for logic models to prevent starting from scratch in each project.

### Strengths and Limitations

This scoping review provides, to our knowledge, the first systematic mapping of how logic models and ToCs are used within HIT research. By analyzing 69 peer-reviewed studies across diverse disciplines, the review offers a comprehensive overview of the purposes and scope of HIT logic models. A strength lies in the systematic and transparent data extraction process, which covered not only descriptive features but also visual and theoretical aspects of each logic model.

Some limitations must, however, be acknowledged. First, although major bibliographic databases were searched, inclusion was restricted to peer-reviewed, English-language publications, and gray literature was not systematically explored. Because logic models are often used in program management and development cooperation, additional project reports may exist outside the scientific literature. Nevertheless, it is unlikely that inclusion of such sources would have altered the main findings of this review, as our analysis focused on scoping the nature rather than the frequency of logic model use.

Second, the heterogeneity of reporting limited the depth of analysis. Many studies did not provide sufficient detail on model development or theoretical basis. No validated appraisal tool currently exists for assessing the quality of logic models, making it difficult to distinguish between well-grounded models and more schematic representations.

Finally, the review reflects a snapshot of the literature up to 2025. Given the rapid evolution of HIT and the increasing integration of implementation science frameworks, future studies may show substantial changes in how logic models are constructed and applied. Continuous updating and cross-field comparison will therefore be essential to monitor progress toward methodological standardization and deeper theoretical integration.

### Comparison With Other Fields

The use of program theory and logic models is well-established across several health and social science domains, as reflected in the range of guidelines and frameworks identified in this review. For health informatics, however, only 2 specific frameworks – FITT and NASSS – were identified.

In public health, logic models and theories of change have been a standard requirement for designing, implementing, and evaluating complex interventions [3]. They are embedded in national and international frameworks, such as those of the Centers for Disease Control and Prevention [98], the World Health Organization [93], and the UK NICE [99], all of which emphasize explicit articulation of causal assumptions and outcome chains. In contrast, comparable guidance for HIT remains scarce. Besides, HIT interventions often focus on evaluating short-term metrics or user acceptance measures, with limited attention to underlying mechanisms of change, long-term outcomes, or system-level effects [100].

In implementation science, logic models have evolved into comprehensive meta-frameworks that link determinants, strategies, mechanisms, and outcomes. Frameworks such as the IRLM and the CFIR are now widely adopted to integrate evidence and guide evaluation across multiple levels of health systems. Comparable developments in health informatics or HIT-specific adaptations are not yet visible.

In HTA, logic models have been promoted as a backbone for evidence synthesis and for structuring complex evaluation questions [101,102]. In contrast, few HIT-focused systematic reviews currently apply logic models; in our scoping review, only 6 such reviews were identified. For example, 1 paper on patient decision aids developed several CMO models to explain why and how these tools were successfully implemented in routine care [81].

Overall, program theory from other fields is increasingly used for HIT, but HIT-specific methodological development remains nascent. Only since around 2020 has the systematic use of program theory become visible, but HIT-specific methodological guidance or reporting standards have yet to be developed.

### Future Research Directions

Going forward, advancing the use of logic models in HIT research requires a shift from representation toward explanation and learning. This can be achieved by embedding logic models within evidence-informed development and evaluation cycles, where theoretical and empirical findings feed back into model refinement. Integration with mixed methods designs and implementation frameworks, such as CFIR, IRLM, or NASSS, can help link HIT interventions to contextual factors, thereby helping to achieve the desired outcome of the intervention [91]. Stakeholder-inclusive and iterative model development – involving users, clinicians, managers, and policymakers from the outset – should be promoted as a best practice to ensure that models remain both valid and actionable.

Future work should also address the need for standardization and transparency. Reporting guidelines analogous to STARE-HI [97] could be expanded to require explicit reporting of program theory for HIT evaluations, ensuring consistent description of logic model structure, purpose, and development process. For policy, funding agencies and evaluators should consider making explicit program theory or logic model articulation a requirement for HIT projects, aligning expectations for design, monitoring, and impact assessment.

Another promising direction involves the use of computational and AI-assisted tools to support logic model construction and analysis. Natural language processing and machine learning techniques could assist researchers in extracting components and causal relationships from qualitative data or literature, automatically populating logic model structures, and visualizing linkages between intervention components and outcomes. Such tools would not replace human interpretation but could enhance efficiency, reproducibility, and scalability of program theory development.

Finally, capacity building and education are essential. Educational guidelines in medical informatics, such as the International Medical Informatics Association Recommendations on Health and Medical Education [103], could integrate program theory throughout the HIT innovation lifecycle in their curriculum recommendations.

## Supplementary material

10.2196/87848 Multimedia Appendix 1 Search query.

10.2196/87848 Multimedia Appendix 2 Data extraction of 69 studies.

10.2196/87848 Multimedia Appendix 3 Frameworks and guidelines.

10.2196/87848 Checklist 1 PRISMA-SCR Checklist.

## Abbreviations

CFIR: Consolidated Framework for Implementation Research
CMO: context-mechanism-outcome
COM-B: Capability, Opportunity, Motivation–Behavior model
EHR: electronic health record
ERIC: Expert Recommendations for Implementing Change
FITT: Fit between Individuals, Task and Technology
HIT: health information technology
HTA: health technology assessment
IRLM: implementation research logic model
mHealth: mobile health
MRC: Medical Research Council
NASSS: Non-adoption, Abandonment, and challenges to the Scale-up, Spread, and Sustainability
PCC: Population Concept Context
PRISMA-ScR: Preferred Reporting Items for Systematic Reviews and Meta-Analyses extension for Scoping Reviews
STARE-HI: Standards for Reporting Health Informatics
ToC: theory of change
UTAUT: Unified Theory of Acceptance and Use of Technology
