# Supplementary material for: Logic Models on Health Information Technology–Related Interventions: Scoping Review Across Disciplines
Source: JMIR Med Inform. 2026 Jul 28;14:e87848. doi: 10.2196/87848 (PMC13411438; doi:10.2196/87848)
Supplement: Multimedia Appendix 1 [file medinform-v14-e87848-s001.docx]

Logic Models on Health Information Technology-Related Interventions: A Scoping Review Across Disciplines

Elske Ammenwerth, PhD, UMIT TIROL – Private University for Health Sciences and Health Technology, Hall in Tirol, Austria, ORCID 0000-0002-3244-6918

Michelle Bindel, MSc, UMIT TIROL – Private University for Health Sciences and Health Technology, Hall in Tirol, Austria, ORCID 0000-0002-9383-9678

Iiris Hörhammer, PhD, Aalto University, Finland, ORCID 0000-0001-9024-4153

Corresponding author:

Elske Ammenwerth, Institute of Medical Informatics, UMIT TIROL – Private University for Health Sciences and Health Technology, Eduard Wallnöer Zentrum 1, 6060 Hall in Tirol, Austria, [elske.ammenwerth@umit-tirol.at](mailto:elske.ammenwerth@umit-tirol.at)

# Multimedia Appendix of supplementary files

## Appendix 1: PubMed Query

(("logic model*" OR "results chain" OR "programme logic" OR "causal model" OR "outcome model" OR "logical framework*" OR "logic framework" OR "program logic" OR "theory of change" OR "program theory" OR "results framework" OR "impact pathway")

AND

("Medical Informatics"[MeSH] OR "Health Information Systems"[MeSH] OR "Telemedicine"[MeSH] OR "health information technolog*" OR "Electronic Health Records"[MeSH] OR "Decision Support Systems, Clinical"[MeSH] OR "health informatics" OR "HIT" OR "eHealth" OR "digital health" OR "clinical decision support" OR "mHealth")

AND

("Program Evaluation"[MeSH] OR "Implementation Science"[MeSH] OR evaluation OR implementation OR design OR development OR planning OR "intervention design")

AND

("Health Services"[MeSH] OR "Delivery of Health Care"[MeSH] OR healthcare OR "health care" OR "social care" OR "community care" OR "long-term care" OR "Continuity of Patient Care"[MeSH] OR "Patient Care Management"[MeSH] OR "Delivery of Health Care, Integrated"[MeSH] OR "Primary Health Care"[MeSH] OR "Home Care Services"[MeSH]))

## Appendix 2: List of included papers

Adedeji T, Fraser H, Scott P. Implementing Electronic Health Records in Primary Care Using the Theory of Change: Nigerian Case Study. JMIR Med Inform. 2022;10(8):e33491. doi:10.2196/33491.

Adedeji T, Targema-Takema R, Griffiths A, Scott P. Theory of Change: A Tool for Engaging the Health and Care Workforce in Developing Digital Skills. Stud Health Technol Inform. 2022;298:102-106. doi:10.3233/SHTI220916.

Ali Q, Heldal I, Helgesen CG, Krumina G, Tvedt MN. Technologies supporting vision screening: A protocol for a scoping review. BMJ Open. 2021;11(9):e050819. doi:10.1136/bmjopen-2021-050819.

Andison M. Digital Health Safety Matters: A Promising Practice Study into the Adoption of Patient Safety Guidelines in Australia. Stud Health Technol Inform. 2024;310:334-338. doi:10.3233/SHTI230982.

Aworinde J, Evans CJ, Gillam J, Ramsenthaler C, Davies N, Ellis-Smith C. Co-design of the EMBED-Care Framework as an intervention to enhance shared decision-making for people affected by dementia and practitioners: A qualitative study. Health Expect. 2024;27(1):1-13. doi: 10.1111/hex.13987

Babbage C. Development of a Self-help Digital Intervention for Young People with Tourette Syndrome [dissertation]. Nottingham (UK): University of Nottingham; 2021.

Band R, Bradbury K, Morton K, May C, Michie S, Mair FS, Murray E, McManus RJ, Little P, Yardley L. Intervention planning for a digital intervention for self-management of hypertension: A theory-, evidence- and person-based approach. Implement Sci. 2017;12(1):25. doi:10.1186/s13012-017-0553-4.

Bindel M, Kreyer C, Ammenwerth E. Extracting Evidence from Health IT Studies to Populate Logic Models. Stud Health Technol Inform. 2024;316:207-211. doi:10.3233/SHTI240380.

Bradshaw A, Allsop MJ, Birtwistle J, et al. Exploring the contextual assumptions, interventions and outcomes of digital advance care planning systems: A theory of change approach to understand implementation and evaluation. Palliat Med. 2024;38(10):1144-1155. doi: 10.1177/0269216324128013.

Bramley G, Mangan C, Conroy M. Using telemonitoring to support personal care planning for adults with learning disabilities. J Telemed Telecare. 2019;25(10):602-610. doi:10.1177/1357633X18784419.

Breton M, Smithman MA, Liddy C, et al. Scaling up eConsult for access to specialists in primary healthcare across four Canadian provinces: Study protocol of a multiple case study. Health Res Policy Syst. 2019;17(1):83. doi:10.1186/s12961-019-0483-5.

Busetto L, Luijkx K, Huizing A, Vrijhoef B. Implementation of integrated care for diabetes mellitus type 2 by two Dutch care groups: A case study. BMC Fam Pract. 2015;16(1):105. doi:10.1186/s12875-015-0320-z.

Cen ZF, Tang PK, Hu H, et al. Systematic literature review of adopting eHealth in pharmaceutical care during COVID-19 pandemic: Recommendations for strengthening pharmacy services. BMJ Open. 2022;12(11):e066246. doi:10.1136/bmjopen-2022-066246.

Chaney SC, Mechael P, Thu NM, Diallo MS, Gachen C. Every child on the map: A theory of change framework for improving childhood immunization coverage and equity using geospatial data and technologies. J Med Internet Res. 2021;23(8):e29759. doi:10.2196/29759.

Cross S, Nicholas J, Mangelsdorf S, et al. Developing a Theory of Change for a Digital Youth Mental Health Service (Moderated Online Social Therapy): Mixed Methods Knowledge Synthesis Study. JMIR Form Res. 2023;7(1):e49846. doi:10.2196/49846.

da Costa MP. An Intervention to Connect Patients With Psychosis and Volunteers via Smartphone (the Phone Pal): Development Study. JMIR Form Res. 2022;6(6):e35086. doi:10.2196/35086.

d’Offay C, Ng XY, Alexander L, et al. A Digital Health Intervention for Concussion: Development and Clinical Feasibility Study. JMIR Form Res. 2023;7:e43557. doi:10.2196/43557.

Dohmen P, De Sanctis T, Waiyaiya E, et al. Implementing value-based healthcare using a digital health exchange platform to improve pregnancy and childbirth outcomes in urban and rural Kenya. Front Public Health. 2022;10:1040094. doi:10.3389/fpubh.2022.1040094.

Dolan SB, Wittenauer R, Shearer JC, et al. Integration of a Digital Health Intervention Into Immunization Clinic Workflows in Kenya: Qualitative Realist Evaluation of Technology Usability. JMIR Form Res. 2023;7:e39775. doi:10.2196/39775.

Dutt P, Godfrey A, Chamberlain S, Mitra R. Using behavioural design and theories of change to integrate communication solutions into health systems in India: Evolution, evidence and learnings from practice. Integr Healthc J. 2022;4(1):e000139. doi:10.1136/ihj-2022-000139.

Edwards EA, Caton H, Lumsden J, et al. Creating a theoretically grounded, gamified health app: Lessons from developing the Cigbreak smoking cessation mobile phone game. JMIR Serious Games. 2018;6(4):e10252. doi:10.2196/10252.

Foster CC, Steltzer M, Snyder A, et al. Integrated Multimodality Telemedicine to Enhance In-Home Care of Infants During the Interstage Period. Pediatr Cardiol. 2021;42(2):349-360. doi:10.1007/s00246-020-02489-7.

Ganjali R, Ghorban Sabbagh M, Eslami S. A patient-centered interactive voice response system for supporting self-management in kidney transplantation: Design and field testing. Front Digit Health. 2025;6:1386012. Ddi: 10.3389/fdgth.2024.1386012

Gasteiger N, Van der Veer SN, Wilson P, Dowding D. How, for whom, and in which contexts or conditions augmented and virtual reality training works in upskilling health care workers: Realist synthesis. JMIR Serious Games. 2022;10(1):e31644. doi:10.2196/31644.

Geraghty A, Munoz R, Yardley L, Sharry J, Little P, Moore M. Developing an unguided internet intervention for emotional distress in primary care patients: Applying common factor and person-based approaches. JMIR Ment Health. 2016;3(4):e5845. doi:10.2196/mental.5845.

Hurtubise K, Pratte G, Hamel C, Clapperton I, Camden C. Rethinking early intervention rehabilitation services for children with motor difficulties: Engaging stakeholders in the conceptualization of telerehabilitation primary care. Disabil Rehabil. 2022;44(22):6749-6758. doi:10.1080/09638288.2021.1972173.

Ilozumba O, Dieleman M, Kraamwinkel N, Van Belle S, Chaudoury M, Broerse JEW. “I am not telling, the mobile is telling”: Factors influencing the outcomes of a community health worker mHealth intervention in India. PLoS One. 2018;13(3):e0194927. doi:10.1371/journal.pone.0194927.

Indraratna P, Biswas U, Liu H, et al. Process evaluation of a randomised controlled trial for TeleClinical Care, a smartphone-app based model of care. Front Med. 2022;8:780882. doi:10.3389/fmed.2021.780882.

Ingersoll B, Frost KM, Straiton D, Ramos AP, Casagrande K. Telehealth coaching in Project ImPACT indirectly affects children’s expressive language ability through parent intervention strategy use and child intentional communication: An RCT. Autism Res. 2024;17(10):2177-2187. doi:10.1002/aur.3230.

Israel AMC, van Lenthe FJ, Beenackers MA. A preventive integrated eHealth approach for individuals with a low socioeconomic position: Protocol for a realist evaluation. BMC Public Health. 2024;24(1):2700. doi:10.1186/s12889-024-20113-8.

Joseph-Williams N, Abhyankar P, Boland L, et al. What works in implementing patient decision aids in routine clinical settings? A rapid realist review and update from the International Patient Decision Aid Standards Collaboration. Med Decis Making. 2021;41(7):907-937. doi:10.1177/0272989X20978208.

Kang B, Chin L, Camacho-Rivera M, et al. Intervention mapping for systematic development of a community-engaged CVD prevention intervention in ethnic and racial sexual minority men with HIV. Front Public Health. 2025;13:1529152. doi:10.3389/fpubh.2025.1529152.

Korpershoek YJG, Hermsen S, Schoonhoven L, Schuurmans MJ, Trappenburg JCA. User-centered design of a mobile health intervention to enhance exacerbation-related self-management in patients with chronic obstructive pulmonary disease (Copilot): Mixed methods study. J Med Internet Res. 2020;22(6):e15449. doi:10.2196/15449.

Krasuska M, Davidson EM, Beune E, et al. A culturally adapted diet and physical activity text message intervention to prevent type 2 diabetes mellitus for women of Pakistani origin living in Scotland: Formative study. JMIR Form Res. 2023;7(1):e33810. doi:10.2196/33810.

Lei KC, Loi CI, Cen Z, et al. Adopting an electronic medication administration system in long-term care facilities: A key stakeholder interview study in Macao. Inform Health Soc Care. 2023;48(3):252-266. doi:10.1080/17538157.2023.2165084.

Marques MM, Matos M, Mattila E, et al. A theory- and evidence-based digital intervention tool for weight loss maintenance (NoHoW Toolkit): Systematic development and refinement study. J Med Internet Res. 2021;23(12):e25305. doi:10.2196/25305.

McLaughlin P, Hurley M, Chowdary P, Khair K, Smith C, Stephensen D. Using theory of change to co-create a programme theory for a telerehabilitation intervention for pain management in people with haemophilia. Orphanet J Rare Dis. 2023;18(1):376. doi:10.1186/s13023-023-02988-9.

Meiksin R, Melendez-Torres GJ, Falconer J, Witzel TC, Weatherburn P, Bonell C. Theories of change for e-health interventions targeting HIV/STIs and sexual risk, substance use and mental ill health amongst men who have sex with men: Systematic review and synthesis. Syst Rev. 2021;10(1):21. doi:10.1186/s13643-020-01523-2.

Millard T, Dodson S, McDonald K, et al. The systematic development of a complex intervention: HealthMap, an online self-management support program for people with HIV. BMC Infect Dis. 2018;18(1):615. doi:10.1186/s12879-018-3518-6.

Millery M, Ramos W, Lien C, Aguirre AN, Kukafka R. Design of a community-engaged health informatics platform with an architecture of participation. AMIA Annu Symp Proc. 2015;2015:905-914.

Morgan B, Hunt X, Tomlinson M. Thinking about the environment and theorising change: How could Life History Strategy Theory inform mHealth interventions in low- and middle-income countries? Glob Health Action. 2017;10(1):1320118. doi:10.1080/16549716.2017.1320118.

Mukherjee A, Yatirajula SK, Kallakuri S, et al. Using formative research to inform a mental health intervention for adolescents living in Indian slums: The ARTEMIS study. Child Adolesc Psychiatry Ment Health. 2024;18(1):1-14. doi: 10.1186/s13034-024-00704-4.

Nigatu D, Azage M, Misgan E, et al. Implementation research logic model in the design and execution of eHealth innovations for maternal and newborn healthcare in Ethiopia. Health Res Policy Syst. 2025;23(1):4. doi:10.1186/s12961-024-01259-8.

Nupdal J. Implementing clinical practice guidelines in family practice: Caring for children with ADHD [dissertation]. Fargo (ND): North Dakota State University; 2014.

Orchard J, Li J, Gallagher R, Freedman B, Lowres N, Neubeck L. Uptake of a primary care atrial fibrillation screening program (AF-SMART): A realist evaluation of implementation in metropolitan and rural general practice. BMC Fam Pract. 2019;20(1):170. doi:10.1186/s12875-019-1058-9.

Palacz-Poborczyk I, Idziak P, Januszewicz A, et al. Developing the “Choosing Health” digital weight loss and maintenance intervention: Intervention mapping study. J Med Internet Res. 2022;24(10):e34089. doi:10.2196/34089.

Patel AB, Kuhite PN, Khan SS, et al. M-SAKHI—Mobile health solutions to help community providers promote maternal and infant nutrition and health: A description of development of the Program Impact Pathway using Theory of Change. Public Health Nutr. 2024;27(1):e217. doi:10.1017/S1368980024001265.

Peprah P, Osman S, Mitchell R, et al. Outpatient virtual care among people living with and beyond cancer from culturally and linguistically diverse backgrounds in Australia: A protocol for a realist evaluation. Health Expect. 2025;28(2):e70235. doi:10.1111/hex.70235.

Pickel J, Fiks AG, Karavite D, et al. Provider perspectives on clinical decision support to improve HIV prevention in pediatric primary care: A multiple methods study. Implement Sci Commun. 2023;4(1):18. doi:10.1186/s43058-023-00394-7.

Pyron L, Carterleton H. Improved patient flow and provider efficiency after the implementation of an electronic health record. Comput Inform Nurs. 2019;37(10):513-521. doi:10.1097/CIN.0000000000000553.

Rambach T, Kunze C. Co-designing AI interventions: A participatory approach using system mapping and Theory of Change modelling. Stud Health Technol Inform. 2025;327:400-401. doi:10.3233/SHTI250356.

Richards R, Jones RA, Whittle F, et al. Development of a web-based, guided self-help, acceptance and commitment therapy–based intervention for weight loss maintenance: Evidence-, theory-, and person-based approach. JMIR Form Res. 2022;6(1):e31801. doi:10.2196/31801.

Seymour B, Hackley D, Muriithi M, et al. Developing a WHO African Region mOral Health Curriculum for Community Health Workers. Ann Glob Health. 2025;91(1):1-13. doi:10.5334/aogh.4655.

Shegog R, Begley CE. Clinic-based mobile health decision support to enhance adult epilepsy self-management: An intervention mapping approach. Front Public Health. 2017;5:256. doi:10.3389/fpubh.2017.00256.

Silva IS, Silva CRDV, Martiniano CS, et al. Digital health and quality of care in primary health care: An evaluation model. Front Public Health. 2024;12:1443862. doi:10.3389/fpubh.2024.1443862.

Simons M, Harvey G, McMillan L, et al. Implementation outcomes of a digital, trauma-informed care, educational intervention targeting health professionals in a paediatric burns setting: A mixed methods process evaluation. Burns. 2024;50(6):1690-1703. doi:10.1016/j.burns.2024.03.032.

Söling S, Demirer I, Köberlein-Neu J, et al. Complex implementation mechanisms in primary care: Do physicians’ beliefs about the effectiveness of innovation play a mediating role? Applying a realist inquiry and structural equation modeling approach in a formative evaluation study. BMC Prim Care. 2023;24(1):131. doi:10.1186/s12875-023-02081-x.

Sousa JL, Raja P, Rabinowitz M, et al. Patient experiences with group teletherapy for the treatment of mental illness: A qualitative study. Psychiatr Serv. 2025;76(2):157-162. doi:10.1176/appi.ps.20240058.

Strumann C, Pfau L, Wahle L, Schreiber R, Steinhäuser J. Designing and implementation of a digitalized intersectoral discharge management system and its effect on readmissions: Mixed methods approach. J Med Internet Res. 2024;26(1):e47133. doi:10.2196/47133.

Taylor AH, Taylor RS, Ingram WM, et al. Adding web-based behavioural support to exercise referral schemes for inactive adults with chronic health conditions: The e-coacher RCT. Health Technol Assess. 2020;24(63):1-105. doi:10.3310/hta24630.

Teriö M, Eriksson G, Kamwesiga JT, Guidetti S. What’s in it for me? A process evaluation of the implementation of a mobile phone-supported intervention after stroke in Uganda. BMC Public Health. 2019;19(1):562. doi:10.1186/s12889-019-6849-3.

Terp R, Lindhardt T, Kayser L. Theory-driven development of an educative nutritional intervention supporting older hospital patients to eat sufficiently, assisted by an eHealth solution: An intervention mapping approach. BMC Health Serv Res. 2022;22(1):1435. doi:10.1186/s12913-022-08679-8.

Thomson M, Henderson G, Rogers T, et al. Digital mental health and peer support: Building a Theory of Change informed by stakeholders’ perspectives. PLoS Digit Health. 2024;3(5):e0000522. doi:10.1371/journal.pdig.0000522.

Voorwinde V, Steenhuis IHM, Janssen IMC, Monpellier VM, van Stralen MM. Getting back on track: Development of a blended-care intervention for weight recurrence after metabolic bariatric surgery using intervention mapping. PEC Innov. 2025;6:100404. doi:10.1016/j.pecinn.2025.100404.

Vugts MAP, Zedlitz AMEE, Joosen MCW, Vrijhoef HJM. Serious gaming during multidisciplinary rehabilitation for patients with chronic pain or fatigue symptoms: Mixed methods design of a realist process evaluation. J Med Internet Res. 2020;22(3):e14766. doi:10.2196/14766.

Walker DM, Hefner JL, Sieck CJ, Huerta TR, McAlearney AS. Framework for evaluating and implementing inpatient portals: A multi-stakeholder perspective. J Med Syst. 2018;42(9):155. doi:10.1007/s10916-018-1009-3.

Wan TTH, Wang BL. An integrated social and behavioral system approach to evaluation of healthcare information technology for polychronic conditions. J Integr Des Process Sci. 2021;25(3-4):148-160.

Wang R, Rouleau G, Booth GL, et al. Understanding whether and how a digital health intervention improves transition care for emerging adults living with type 1 diabetes: Protocol for a mixed methods realist evaluation. JMIR Res Protoc. 2023;12:e46115. doi:10.2196/46115.

Wheldon CW, Grace J, Zimet G, et al. Development and evaluation of a decision aid for HPV vaccination among adults aged 27–45 years old in the United States. Comput Biol Med. 2025;185:109557. doi:10.1016/j.compbiomed.2024.109557.

## Appendix 3: Full summary table

<separate Shee>

## Appendix 4: Glossary of Theories and Frameworks

To allow for a more precise analysis of the theoretical foundations of logic models and theories of change, we differentiated between two types of theoretical guidance observed in the included studies. First, we extracted the guidelines and frameworks on how to develop a logic model cited in the included papers. Second, we extracted frameworks and theories for populating the logic model cited in the papers.

To see which papers used each theory or framework, refer to Appendix 4.

In the following two sections, we will use the following presentation template:

| Abbreviation | Long name of the guideline, theory or framework |
| --- | --- |
| Explanation – what is the guideline, theory or framework all about? | |
| Main literature sources referenced in the included papers | |

### Guidelines and frameworks on how to develop a logic model

| Baxter et al. | Baxter et al. Logic model synthesis approach to Systematic Reviews |
| --- | --- |
| Baxter et al. (2014) developed a method that integrates systematic review techniques with logic model synthesis to map how complex interventions lead to outcomes. | |
| Baxter SK, Blank L, Woods HB, Payne N, Rimmer M, Goyder E. Using logic model methods in systematic review synthesis: describing complex pathways in referral management interventions. BMC Med Res Methodol. (2014) 14(1):1–9. doi: 10.1186/1471-2288-14-62 | |

| Breuer et al. | Breuer et al. Overview of Logic Model use in Public health |
| --- | --- |
| In their 2016 systematic review, Breuer, Lee, De Silva, and Lund show that ToC is increasingly used in public health to articulate causal pathways, guide data collection, and integrate evidence, ultimately enhancing the design and assessment of complex interventions. | |
| Breuer E, De Silva M, Lund C. Theory of change for complex mental health interventions: 10 lessons from the programme for improving mental healthcare. Global Mental Health. 2018;5. https://doi.org/10.1017/gmh.2018.13 PMID: 30128160  Breuer E, Lee L, De Silva M, Lund C. Using theory of change to design and evaluate public health interventions: A systematic review. Implementation Science. 2016; 11(1).<https://doi.org/10.1186/s13012-> 016-0422-6 PMID: 27153985 | |

| CDC | Centers for Disease Control and Prevention |
| --- | --- |
| The CDC’s framework and guides collectively provide a structured approach to public health program evaluation, outlining clear steps and tools for planning, implementing, and assessing programs. | |
| Centers for Disease Control and Prevention (1999) Framework for program evaluation in public health. Morbidity Mortality Weekly Report Recommendations Rep. 48(Rr-11):1–40.  CDC. CDC-Division for heart disease and stroke prevention evaluation guide: developing and using a logic model. Atlanta, GA: CDC, 2006  Centers for Disease Control and Prevention. Logic Models—A CDC Framework Approach to Program Evaluation. CDC; Published December 18, 2018. | |

| Coffmann | Coffmann Framework for Program evaluation |
| --- | --- |
| This research brief shows how to develop and use a logic model as a framework for an evaluation of a program or an organization. | |
| Coffman, J. (1999). Harvard Family Research Project. Learning from logic models: An example of a family/school partnership program. Retrieved from www.hfrp.org | |

| Donabedian | Donabedian Health Care Quality Evaluation Framework |
| --- | --- |
| Donabedian’s works (1980, 2005) present a foundational framework for assessing healthcare quality based on three key components: structure, process, and outcome. | |
| Donabedian A. Basic approaches to assessment: Structure, process and outcome. Donabedian A. Explorations in Quality Assessment and Monitoring 1:75–125, 1980  Donabedian A. Evaluating the quality of medical care. Milbank Q. (2005) 83:691–729. doi: 10.1111/j.1468-0009.2005.00397.x | |

| i-PARIHS | Integrated Promoting Action on Research Implementation in Health Services |
| --- | --- |
| The i-PARIHS (integrated Promoting Action on Research Implementation in Health Services) framework builds on the original PARIHS model to better explain and predict the success of evidence implementation in healthcare. | |
| Harvey G, Kitson A. PARIHS revisited: from heuristic to integrated framework for the successful implementation of knowledge into practice. Implement Sci. 2016 Mar 10;11:33. https://doi.org/10.1186/s13012-016-0398. | |

| IRLM | Implementation Research Logic Model |
| --- | --- |
| The Implementation Research Logic Model (IRLM) provides a structured framework for planning, executing, and evaluating implementation research. It helps researchers clearly articulate how implementation strategies are expected to produce change, improving the transparency, rigor, and reproducibility of implementation studies. | |
| Smith JD, Li DH, Rafferty MR. The Implementation Research Logic Model: a method for planning, executing, reporting, and synthesizing implementation projects. Implementation Sci. (2020) 15:84. doi:10.1186/s13012-020-01041-8 | |

| Joly et al. | Joly et al. Framework on Public Health Outcomes |
| --- | --- |
| The article explores the role of accreditation and performance standards in improving public health systems and outcomes, highlighting the current lack of empirical evidence linking accreditation to population health improvement. It proposes a logic model framework to clarify how accreditation processes influence organizational and community outcomes, guiding future research, measurement development, and evaluation efforts in public health accreditation. | |
| Joly, B. M., Polyak, G., Davis, M. V., Brewster, J., Tremain, B., Raevsky, C., & Beitsch, L. M. (2007). Linking Accreditation and Public Health Outcomes. Journal of Public Health Management and Practice, 13(4), 349–356. https://doi.org/10.1097/01.PHH.0000278027.56820.7e. | |

| Kellogg | W.K. Kellogg Foundation Logic Model Development Guide |
| --- | --- |
| The W.K. Kellogg Foundation Logic Model Development Guide offers practical guidance on creating and using logic models to plan, implement, and evaluate programs. | |
| W.K. Kellogg Foundation. W.K. Kellogg Foundation Logic Model Development Guide. 2004.  Knowlton, L. W., & Phillips, C. C. (2012). The logic model guidebook: Better strategies for great results. Sage. | |

| LIFE | Local Initiatives for Faith and Evaluation |
| --- | --- |
| The LIFE Framework (Local Initiatives for Faith and Evaluation), developed by the Australian Government Department of Health and Ageing is a planning and evaluation framework designed to support community-based health and wellbeing initiatives. | |
| Mitchell P, Lewis V. A Manual to Guide the Development of Local Evaluation Plans: Evaluating Initiatives within the LIFE Framework Using a Program Logic Approach. Canberra: Australian Government Department of Health and Aging; 2003. | |

| MRC | Medical Research Council guidance |
| --- | --- |
| The MRC (Medical Research Council) guidance provides a comprehensive framework for developing and evaluating complex interventions in health and social care. | |
| Moore GF, Audrey S, Barker M et al. Process evaluation of complex interventions: Medical Research Council guidance. BMJ. 2015;350:h1258. PMID: 25791983  Craig P, Dieppe P, Macintyre S, Michie S, Nazareth I, Petticrew M. Developing and evaluating complex interventions: the new Medical Research Council guidance. BMJ. 2008;337.  De Silva MJ, Breuer E, Lee L, Asher L, Chowdhary N, Lund C, et al. Theory of Change: a theory-driven approach to enhance the Medical Research Council's framework for complex interventions. Trials 2014 Jul 05;15:267 [FREE Full text] [doi:10.1186/1745-6215-15-267] [Medline: 24996765]  Skivington K, Matthews L, Simpson SA, et al. A new framework for developing and evaluating complex interventions: update of Medical Research Council guidance. BMJ. 2021;374:n2061. | |

| PBRN | Practice-based research network |
| --- | --- |
| Hayes, Parchman, and Howard (2011) present a logic model framework designed to support evaluation and planning within primary care practice-based research networks (PBRNs). | |
| Hayes H, Parchman ML, Howard R. A logic model framework for evaluation and planning in a primary care practice-based research network (PBRN). J Am Board Fam Med. 2011;24:576-582. PMID: 21900441 | |

| PSM | Participatory Systems Mapping |
| --- | --- |
| Wilkinson, Hills, Penn, and Barbrook-Johnson (2021) present a method for developing a system-based Theory of Change (ToC) using Participatory Systems Mapping to capture the complexity of social programs. Their approach integrates stakeholder perspectives to visualize dynamic relationships, feedback loops, and interdependencies within systems, leading to more comprehensive and adaptive program design and evaluation. | |
| Wilkinson H, Hills D, Penn A, Barbrook-Johnson P. Building a system-based Theory of Change using Participatory Systems Mapping. Evaluation. 2021;27(1):80–101.  doi:10.1177/1356389020980493 | |

| Realist Evaluation | Realist evaluation |
| --- | --- |
| These works collectively advance the realist evaluation approach, which seeks to explain how, why, for whom, and under what conditions interventions work by analyzing the interaction of context, mechanisms, and outcomes (CMO configurations). | |
| Pawson R, Tilley N. Thousand Oaks: Realist Evaluation. Sage Publications. 1997.  Greenhalgh J, Manzano A. Understanding ‘context’ in realist evaluation and synthesis. Int J Soc Res Methodol. 2022;25:583–95. https://doi. org/ 10. 1080/ 13645 579. 2021. 19184 84.  Dalkin SM, Greenhalgh J, Jones D, et al. What’s in a mechanism? Development of a key concept in realist evaluation. Implementation Sci. 2015;10:49. https:// doi. org/ 10. 1186/ s13012-​015-​0237-x. | |

| Rogers et al. | Rogers et al. TOC approach with a focus on complex interventions |
| --- | --- |
| Rogers explains how program theory and Theory of Change approaches can be used to evaluate complicated and complex interventions by mapping the causal pathways between activities and outcomes. | |
| Rogers PJ. Using programme theory to evaluate complicated and complex aspects of interventions. Evaluation. 2008;14(1):29–48.  Rogers P (2014) Theory of Change: Methodological Briefs - Impact Evaluation No. 2. New York: UNICEF  Funnell SC and Rogers PJ. Purposeful program theory: effective use of theories of change and logic models. San Francisco, USA: John Wiley & Sons, 2011. | |

| UKDFID | UK Department for International Development |
| --- | --- |
| The UK DFID’s Theory of Change approach, based on Vogel’s 2012 review, provides a structured method for mapping how and why desired changes are expected to occur within complex development programs. | |
| Review of the use of ‘Theory of Change’ in international development. UK Department of International Development.  Vogel I. Review of the use of “Theory of Change” in international development Review Report. 2012  United Nations Development Group (2017) UNDAF CompanionGuidance: Theory of Change. New York: United Nations Sustainable Development Group. | |

### Frameworks and theories for populating the logic model

| CCM | Chronic Care Model |
| --- | --- |
| The Chronic Care Model (CCM) developed by Wagner (1998) provides a framework for improving outcomes in chronic disease management by aligning health system organization, self-management support, delivery system design, decision support, and clinical information systems to create productive interactions between informed patients and proactive healthcare teams. | |
| Wagner EH. Chronic disease management: what will it take to improve care for chronic illness? Eff Clin Pract. 1998;1(1):2–4. | |

| CFIR | Consolidated Framework for Implementation Research |
| --- | --- |
| The Consolidated Framework for Implementation Research (CFIR), developed by Damschroder and colleagues (2009, updated 2022), provides a comprehensive structure for identifying factors that influence the successful implementation of evidence-based interventions across five domains: intervention characteristics, outer setting, inner setting, characteristics of individuals, and implementation process. | |
| Damschroder LJ, Aron DC, Keith RE, et al. Fostering implementation of health services research findings into practice: a consolidated framework for advancing implementation science. Implement Sci. 2009;4(1):50–15.  Damschroder LJ, Reardon CM, Widerquist MAO, Lowery J. The updated consolidated framework for implementation research based on user feedback. Implement Sci 2022;17(1):75, doi: 10.1186/s13012-022-01245-0  Moullin JC, Dickson KS, Stadnick NA, Albers B, Nilsen P, Broder-Fingert  S, et al. Ten recommendations for using implementation frameworks in  research and practice. Implement Sci Commun. 2020;1:42. | |

| COM-B | COM-B model, with Behaviour Change Wheel (BCW) and Theoretical Domains Framework (TDF) |
| --- | --- |
| Michie, West, and colleagues (2008–2016) developed the Behaviour Change Wheel (BCW) as a comprehensive framework for designing and evaluating behavior change interventions. At its core lies the COM-B model - which identifies that behavior (B) results from the interaction of Capability, Opportunity, and Motivation - and around it sits a systematic process for selecting intervention functions and policy categories to target those components.  The Theoretical Domains Framework (TDF) complements COM-B and BCW by breaking down the psychological and contextual factors influencing behavior into 14 detailed domains (e.g., knowledge, skills, beliefs about capabilities, social influences). In essence, COM-B provides the foundational structure of what needs to change, the TDF offers a deeper theoretical understanding of why those factors matter, and the BCW integrates both into a practical tool for designing, implementing, and evaluating effective behavior change interventions. | |
| Michie S, van Stralen MM, West R. The behaviour change wheel: a new method for characterising and designing behaviour change interventions. Implement Sci 2011;6:42 [doi: 10.1186/1748-5908-6-42]  West R, Michie S. A Guide to Development and Evaluation of Digital Behavior Change Interventions in Health Care. London: Silverback Publishing; 2016.  Michie S, Johnston M, Francis J, Hardeman W, Eccles M. From theory to intervention: mapping theoretically derived behavioural determinants to behaviour change techniques. Appl Psychol 2008 Oct;57(4):660-680 [doi: 10.1111/j.1464 0597.2008.00341.x] | |

| CSM | Common Sense Model |
| --- | --- |
| The  extended Common Sense Model (CSM) describes how individuals form cognitive and emotional representations of illness that guide their coping behaviors and self-management. The model explains how people interpret symptoms, choose coping strategies, and adjust behaviors over time through a continuous process of self-regulation aimed at restoring health or well-being. | |
| Leventhal H, Phillips LA, Burns E. The Common-Sense Model of Self-Regulation (CSM): a dynamic framework for understanding illness self-management. J Behav Med. 2016 Dec;39(6):935-946. doi: 10.1007/s10865-016-9782-2. PMID: 27515801. | |

| ERIC | Expert Recommendations for Implementing Change |
| --- | --- |
| The Expert Recommendations for Implementing Change (ERIC) project by Powell, Waltz, and colleagues developed and refined a comprehensive, consensus-based taxonomy of implementation strategies to support the adoption and integration of evidence-based practices. The framework identifies, defines, and organizes discrete strategies, such as training, facilitation, and stakeholder engagement, to help researchers and practitioners select and report methods that effectively drive implementation change. | |
| Powell BJ, Waltz TJ, Chinman MJ, Damschroder LJ, Smith JL, Matthieu MM, et al. A refined compilation of implementation strategies: results from the Expert Recommendations for Implementing Change (ERIC) project. Implement Sci 2015;10:21 [doi: 10.1186/s13012-015-0209-1]  Waltz TJ, Powell BJ, Chinman MJ, Smith JL, Matthieu MM, Proctor EK, et al. Expert recommendations for implementing change (ERIC): protocol for a mixed methods study. Implement Sci. 2014;9(1):39. | |

| FITT | Fit Framework for understanding IT adoption in healthcare |
| --- | --- |
| Ammenwerth, Iller, and Mahler (2006) present a Fit Framework for understanding IT adoption in healthcare, emphasizing the interaction between task, technology, and individual factors. The model proposes that successful adoption occurs when there is an optimal fit among these elements, highlighting how mismatches can hinder effective technology use and implementation in clinical settings. | |
| Ammenwerth E, Iller C, Mahler C. IT-adoption and the interaction of task, technology and individuals: a fit framework and a case study. BMC Med Inform Decis Mak 2006 Jan 09;6:3 [doi: 10.1186/1472-6947-6-3] | |

| Grol et al. | Grol et al. Framework on barriers of evidence-based practice in healthcare |
| --- | --- |
| Grol and Wensing (2004) present a framework for understanding barriers and incentives that influence the adoption of evidence-based practice in healthcare. They identify factors at multiple levels - individual, social, organizational, and systemic - that drive or hinder change, emphasizing the need for tailored strategies to effectively implement and sustain improvements in clinical practice. | |
| Grol R, Wensing M. What drives change? Barriers to and incentives for achieving evidence-based practice. Med J Aust. 2004;180 Suppl 6:57–S60. | |

| HAPA | Health Action Process Approach |
| --- | --- |
| Schwarzer’s Health Action Process Approach (HAPA) is a psychological framework that explains how people adopt and maintain health behaviors through two main phases: motivation (forming intentions) and volition (planning, acting, and maintaining change). It highlights key constructs such as self-efficacy, outcome expectancies, and action planning as drivers of successful behavior change. | |
| Schwarzer R. Modeling health behavior change: how to predict and modify the adoption and maintenance of health behaviors. Appl Psychol 2008;57(1):1-29 [doi: 10.1111/j.1464-0597.2007.00325.x] | |

| HBM | Health Belief Model |
| --- | --- |
| Rosenstock’s Health Belief Model (HBM) explains health behavior through individuals’ perceptions of susceptibility, severity, benefits, barriers, cues to action, and self-efficacy. It posits that people are more likely to engage in preventive or health-promoting behaviors when they believe they are at risk, the consequences are serious, and taking action will effectively reduce that risk. | |
| Rosenstock, I. M. (1966). Why people use health services. In Milbank Quarterly (Vol. 44, Issue 3, pp. 94–124). Blackwell Publishing Inc. https://doi.org/10.1111/j.1468-0009.2005.00425.x.  Rosenstock, I. M., Strecher, V. J., & Becker, M. H. (1988). Social Learning Theory medicineand the Health Belief Model. Health Education & Behavior, 15(2), 175–183. https://doi.org/10.1177/109019818801500203 | |

| Andersen et al | Healthcare Utilization Model |
| --- | --- |
| The Andersen Healthcare Utilization Model explains how predisposing factors, enabling resources, and need influence individuals’ use of health services. It provides a comprehensive framework for understanding healthcare access and utilization by integrating both societal and individual determinants of medical care behavior. | |
| Andersen, R., & Newman, J. F. (1973). Societal and individual determinants of medical care utilization in the United States. Milbank Memorial Fund Quarterly, 51(1), 95–124.https://doi.org/10.2307/3349613.  Babitsch, B., Gohl, D., & von Lengerke, T. (2012). Das Verhaltensmodell der Inanspruchnahme gesundheitsbezogener Versorgung von Andersen re-revisited: Ein systematischer Review von Studien zwischen 1998-2011. In GMS Psycho-Social-Medicine, vol.9. German Medical Science. https://doi.org/10.3205/psm000089. | |

| I-change | I-Change Model |
| --- | --- |
| De Vries’ (2017) I-Change Model provides an integrated framework for understanding and predicting health behavior change by combining elements from various social-cognitive theories. It emphasizes three main phases: awareness, motivation, and action, and highlights how factors such as knowledge, risk perception, self-efficacy, and social influences interact to shape behavior change. | |
| De Vries H. An integrated approach for understanding health behavior; the I-change model as an example. Psychol Behav Sci Int J. 2017;2(2):555–85. | |

| IM | Implementation Mapping |
| --- | --- |
| The Intervention Mapping (IM) framework is a systematic, theory- and evidence-based approach for developing, implementing, and evaluating health promotion programs. It guides planners through six steps (from needs assessment to evaluation) linking behavioral and environmental determinants to practical strategies, and has been extended through Implementation Mapping to design targeted implementation strategies. | |
| Bartholomew LK, Parcel GS, Kok G. Intervention mapping: a process for developing theory- and evidence-based health education programs. Health Educ Behav. Oct 1998;25(5):545-563. [doi: 10.1177/109019819802500502] [Medline: 9768376]  Fernandez ME, Ten Hoor GA, van Lieshout S, Rodriguez SA, Beidas RS, Parcel G, Ruiter RAC, Markham CM, Kok G. Implementation Mapping: Using Intervention Mapping to Develop Implementation Strategies. Front Public Health. 2019 Jun 18;7:158. doi: 10.3389/fpubh.2019.00158. | |

| NASSS | NASSS framework |
| --- | --- |
| Greenhalgh and Abimbola’s (2019) NASSS framework (Nonadoption, Abandonment, Scale-up, Spread, and Sustainability) synthesizes multiple theories to explain the complexity of technology implementation in healthcare. It identifies seven interacting domains, such as the technology, the adopters, the organization, and the wider system, that influence whether digital health innovations are successfully adopted and sustained. | |
| Greenhalgh T and Abimbola S. The NASSS framework – a synthesis of multiple theories of technology implementation. Stud Health Technol Inform 2019; 263: 193–204. | |

| NPT | Normalization process theory |
| --- | --- |
| May (2006) introduced the Normalization Process Theory (NPT) as a framework for understanding how complex interventions become routinely embedded in healthcare practice. It focuses on the collective actions and social processes, such as coherence, cognitive participation, collective action, and reflexive monitoring, that influence successful implementation and integration of new practices. | |
| May C. A rational model for assessing and evaluating complex interventions in health care. BMC Health Serv Res. 2006;6(86). | |

| ODSF | Ottawa Decision Support Framework |
| --- | --- |
| A well-established framework that identifies determinants essential for high-quality medical decisions, particularly for those in which uncertainty needs to be considered. | |
| L. Hoefel, A.M. O’Connor, K.B. Lewis, L. Boland, L. Sikora, J. Hu, D. Stacey, 20th anniversary update of the Ottawa decision support framework Part 1: a systematic review of the decisional needs of people making health or social decisions, Med. Decis. Making 40 (2020) 555–581,https://doi.org/10.1177/0272989X20936209. | |

| Proctor et al. | Proctor et al. Outcome Framework for Implementation Research |
| --- | --- |
| Proctor et al. propose a framework that defines and distinguishes implementation outcomes, such as acceptability, adoption, appropriateness, feasibility, fidelity, cost, penetration, and sustainability. This model clarifies how these outcomes differ from service and clinical outcomes, providing a foundation for measuring and advancing implementation research in health and mental health settings. | |
| Proctor E, Silmere H, Raghavan R, Hovmand P, Aarons G, Bunger A, et al. Outcomes for implementation research: conceptual distinctions, measurement challenges, and research agenda. Adm Policy Ment Health. 2011;38(2):65–76. | |

| READHY | Readiness and Enablement Index for Health Technology (READHY) framework |
| --- | --- |
| Kayser et al. (2019) developed the READHY (Readiness and Enablement Index for Health Technology) tool as a multidimensional framework to assess individuals’ readiness to engage with digital health technologies. It integrates measures of eHealth literacy, self-management, and social support, providing a comprehensive way to evaluate personal and contextual factors influencing technology adoption in healthcare. | |
| Kayser L, Rossen S, Karnoe A, Elsworth G, Vibe-Petersen J, Christensen JF, et al. Development of the multidimensional Readiness and Enablement Index for health Technology (READHY) tool to measure individuals’ health technology readiness: Initial test | |

| SCT | Social cognitive theory |
| --- | --- |
| Bandura’s (1989) Social Cognitive Theory (SCT) explains behavior as the result of dynamic interactions between personal factors, environmental influences, and behavior itself. It highlights key constructs such as observational learning, self-efficacy, and reciprocal determinism, emphasizing that people learn and maintain behaviors through both individual agency and social context. | |
| Bandura A. Human agency in social cognitive theory. Am Psychol. 1989;44: 1175. | |

| SDT | Self determination theory |
| --- | --- |
| Ryan et al. (2008) apply Self-Determination Theory (SDT) to health behavior change, emphasizing that interventions are most effective when they support individuals’ autonomy, competence, and relatedness. The framework explains how fostering intrinsic motivation and self-regulation leads to more sustained engagement in healthy behaviors over time. | |
| Ryan RM, et al. Facilitating health behaviour change and its maintenance: interventions based on self-determination theory. Eur Health Psychol 2008;10(1): 2–5. | |

| SEIPS | Systems Engineering Initiative for Patient Safety |
| --- | --- |
| Holden et al. (2013) present the SEIPS 2.0 (Systems Engineering Initiative for Patient Safety) framework, a human factors and systems-based model for analyzing and improving healthcare work systems. It emphasizes the interactions among people, tasks, tools, technologies, environments, and organizational structures to enhance patient safety, care quality, and staff well-being, a perspective further applied by Or et al. (2014) to understand socio-technical barriers in HIT implementation. | |
| Holden RJ, Carayon P, Gurses AP et al. , SEIPS 2.0: A human factors framework for studying and improving the work of healthcare professionals and patients. Ergonomics 56(11):1669–1686, 2013. 10.1080/00140139.2013.838643.  Or C, Dohan M, and Tan J, Understanding critical barriers to implementing a clinical information system in a nursing home through the lens of a socio-technical perspective. J. Med. Syst. 38(9):99, 2014. 10.1007/s10916-014-0099-9. | |

| TAM | Technology Acceptance Model |
| --- | --- |
| The Technology Acceptance Model (TAM), developed by Davis (1989) and expanded by later researchers such as Venkatesh and Bala (2008), explains users’ acceptance and use of technology based on two key perceptions: usefulness and ease of use. The framework has evolved through multiple versions and empirical studies (e.g., Turner et al., 2010) to better predict technology adoption behaviors and guide the design of user-centered technological interventions. | |
| Davis FD. Perceived usefulness, perceived ease of use, and user acceptance of information technology. MIS Q 1989;13(3):319-340 [doi: 10.2307/249008]  Turner, M., Kitchenham, B., Brereton, P., Charters, S., & Budgen, D. (2010). Does the technology acceptance model predict actual use? A systematic literature review. In Information and Software Technology 52 (5), 463–479. Elsevier. https://doi.org/10.1016/j.infsof.2009.11.005.  Venkatesh V, Bala H. Technology acceptance model 3 and a research agenda on interventions. Decis Sci. 2008;39(2):273–315. | |

| TPB | Theory of planned behaviour |
| --- | --- |
| The Theory of Planned Behavior (TPB) explains how attitudes, subjective norms, and perceived behavioral control shape individuals’ intentions and actions. | |
| Ajzen I. The theory of planned behavior. Organ Behav Hum Decis Process 1991;50 (2):179–211.  Steinmetz H, et al. How effective are behavior change interventions based on the theory of planned behavior? Zeitschrift für Psychol 2016;24(3):216–33. | |

| TTM | Transtheoretical model of health behavior change |
| --- | --- |
| The Transtheoretical Model (TTM) describes health behavior change as a progressive process through five stages: precontemplation, contemplation, preparation, action, and maintenance. | |
| Prochaska JO, Velicer WF. The transtheoretical model of health behavior change. Am J Health Promot. 1997;12:38–48. | |

| UTAUT | Unified Theory of Acceptance and Use of Technology |
| --- | --- |
| Venkatesh, Morris, and Davis (2003) developed the Unified Theory of Acceptance and Use of Technology (UTAUT) to explain technology adoption through four core determinants: performance expectancy, effort expectancy, social influence, and facilitating conditions. The model integrates elements from eight earlier technology acceptance theories to provide a unified framework predicting users’ behavioral intentions and actual technology use. | |
| Venkatesh V, Morris MG, Davis GB. User Acceptance of Information Technology: Toward a Unified View. MIS Quarterly JSTOR. 2003;27(3):425–78. https://doi. org/ 10. 2307/ 30036 540. | |

## Appendix 5: PRISMA-ScR Checklist and Explanation

| **SECTION** | **ITEM** | **PRISMA-ScR CHECKLIST ITEM** | **REPORTED ON PAGE #** |
| --- | --- | --- | --- |
| **TITLE** | | | |
| Title | 1 | Identify the report as a scoping review. | 1 |
| **ABSTRACT** | | | |
| Structured summary | 2 | Provide a structured summary that includes (as applicable): background, objectives, eligibility criteria, sources of evidence, charting methods, results, and conclusions that relate to the review questions and objectives. | 3-4 |
| **INTRODUCTION** | | | |
| Rationale | 3 | Describe the rationale for the review in the context of what is already known. Explain why the review questions/objectives lend themselves to a scoping review approach. | 5-7 |
| Objectives | 4 | Provide an explicit statement of the questions and objectives being addressed with reference to their key elements (e.g., population or participants, concepts, and context) or other relevant key elements used to conceptualize the review questions and/or objectives. | 7 |
| **METHODS** | | | |
| Protocol and registration | 5 | Indicate whether a review protocol exists; state if and where it can be accessed (e.g., a Web address); and if available, provide registration information, including the registration number. | 7 |
| Eligibility criteria | 6 | Specify characteristics of the sources of evidence used as eligibility criteria (e.g., years considered, language, and publication status), and provide a rationale. | 7-8 |
| Information sources* | 7 | Describe all information sources in the search (e.g., databases with dates of coverage and contact with authors to identify additional sources), as well as the date the most recent search was executed. | 8 |
| Search | 8 | Present the full electronic search strategy for at least 1 database, including any limits used, such that it could be repeated. | Appendix 1 |
| Selection of sources of evidence† | 9 | State the process for selecting sources of evidence (i.e., screening and eligibility) included in the scoping review. | 8 |
| Data charting process‡ | 10 | Describe the methods of charting data from the included sources of evidence (e.g., calibrated forms or forms that have been tested by the team before their use, and whether data charting was done independently or in duplicate) and any processes for obtaining and confirming data from investigators. | 8 |
| Data items | 11 | List and define all variables for which data were sought and any assumptions and simplifications made. | 8 |
| Critical appraisal of individual sources of evidence§ | 12 | If done, provide a rationale for conducting a critical appraisal of included sources of evidence; describe the methods used and how this information was used in any data synthesis (if appropriate). | 9 |
| Synthesis of results | 13 | Describe the methods of handling and summarizing the data that were charted. | 9 |
| **RESULTS** | | | |
| Selection of sources of evidence | 14 | Give numbers of sources of evidence screened, assessed for eligibility, and included in the review, with reasons for exclusions at each stage, ideally using a flow diagram. | 10 |
| Characteristics of sources of evidence | 15 | For each source of evidence, present characteristics for which data were charted and provide the citations. | Appendix 4 |
| Critical appraisal within sources of evidence | 16 | If done, present data on critical appraisal of included sources of evidence (see item 12). | n/a |
| Results of individual sources of evidence | 17 | For each included source of evidence, present the relevant data that were charted that relate to the review questions and objectives. | Appendix 4 |
| Synthesis of results | 18 | Summarize and/or present the charting results as they relate to the review questions and objectives. | 10-21 |
| **DISCUSSION** | | | |
| Summary of evidence | 19 | Summarize the main results (including an overview of concepts, themes, and types of evidence available), link to the review questions and objectives, and consider the relevance to key groups. | 22-27 |
| Limitations | 20 | Discuss the limitations of the scoping review process. | 24 |
| Conclusions | 21 | Provide a general interpretation of the results with respect to the review questions and objectives, as well as potential implications and/or next steps. | 27-28 |
| **FUNDING** | | | |
| Funding | 22 | Describe sources of funding for the included sources of evidence, as well as sources of funding for the scoping review. Describe the role of the funders of the scoping review. | 28 |
